# Sleep staging using nocturnal sound analysis

**DOI:** 10.1038/s41598-018-31748-0

**Published:** 2018-09-07

**Authors:** Eliran Dafna, Ariel Tarasiuk, Yaniv Zigel

**Affiliations:** 1 Department of Biomedical Engineering, Faculty of Engineering, Ben-Gurion University of the Negev, Beer–Sheva, Israel; 2 Sleep-Wake Disorders Unit, Soroka University Medical Center, and Department of Physiology, Faculty of Health Sciences, Ben-Gurion University of the Negev, Beer–Sheva, Israel

## Abstract

Sleep staging is essential for evaluating sleep and its disorders. Most sleep studies today incorporate contact sensors that may interfere with natural sleep and may bias results. Moreover, the availability of sleep studies is limited, and many people with sleep disorders remain undiagnosed. Here, we present a pioneering approach for rapid eye movement (REM), non-REM, and wake staging (macro-sleep stages, MSS) estimation based on sleep sounds analysis. Our working hypothesis is that the properties of sleep sounds, such as breathing and movement, within each MSS are different. We recorded audio signals, using non-contact microphones, of 250 patients referred to a polysomnography (PSG) study in a sleep laboratory. We trained an ensemble of one-layer, feedforward neural network classifiers fed by time-series of sleep sounds to produce real-time and offline analyses. The audio-based system was validated and produced an epoch-by-epoch (standard 30-sec segments) agreement with PSG of 87% with Cohen’s kappa of 0.7. This study shows the potential of audio signal analysis as a simple, convenient, and reliable MSS estimation without contact sensors.

## Introduction

Sleep plays a vital role in health and well-being by maintaining health, quality of life, and productivity. Poor sleep is associated with excessive daytime sleepiness, impaired neurocognitive function, increased risk of accidents, and cardiovascular morbidity^1–4^. More than one billion people worldwide exhibit inadequate sleep due to modern lifestyle behavior or physical illness, but most of them are unaware of their sleep disorders^5^, leading to a major challenge for healthcare systems^6^.

Sleep staging is essential for evaluating sleep and its disorders. The main sleep stage categories are S1-S4 and rapid eye movement (REM). Sleep may also be divided into rapid eye movement (REM), non-REM (NREM), and wake (i.e., macro-sleep stages, MSS) activity. Among the most common sleep disorders are insomnia, snoring, and obstructive sleep apnea (OSA)^7^. OSA involves complete or partial breathing cessation during sleep; it is estimated that 10–36% of OSA patients suffer from REM-related OSA, a condition that increases the number of apnea events during REM sleep stage^8^. Identification of REM phases may also assist in predicting other sleep disorders and even mental illnesses. Lately, it was shown that the presence of REM behavior disorder could predict cognitive impairment in Parkinson disease, depression, schizophrenia, mental retardation, and dementia^9–12^.

Sleep stages identification plays a crucial role in determining the quality of sleep, commonly evaluated using polysomnography (PSG) studies^13^. A PSG study, usually performed in sleep laboratories, involves the analysis of electroencephalogram (EEG), electrooculogram (EOG), electromyogram (EMG), and other signals^13^. These physiological signals are measured using many sensors that are connected to the patient, and often may cause discomfort and hence may alter natural sleep and disturb the sleep pattern^14,15^.

Lately, portable monitoring devices are being used to evaluate sleep at-home. These devices offer a reduced-channels approach, such as type 3 portable monitoring, which includes at least four channels such as airflow, respiratory movements, oxyhemoglobin saturation (SpO2), and heart rate^16^. These devices have been utilized as an alternative diagnostic test for sleep disorders^17^ to increase accessibility and to reduce costs. Despite the development of sleep medicine and sleep laboratories, the availability of sleep studies is limited, and many people with sleep disorders remain undiagnosed^6^. Moreover, most type 3 monitors^13^ are designated mainly for diagnosis of obstructive sleep apnea (OSA) and are limited in detecting REM-related OSA or subjects with special needs^18^. Therefore, there is a need for simple and easy to use technology to evaluate sleep and common sleep disorders. Over the past decades, researchers developed algorithms for automatic sleep staging based on EEG signals analysis^19,20^ to ease the tedious procedure of expert analysis and to diminish subjectivity. However, these attempts still use contact sensors that attach to the subject’s head and may disrupt natural sleep.

A variety of physiological changes take place during the different sleep stages^21,22^. It was established that breathing rate becomes regular and decreases on entry into NREM sleep. REM is associated with faster and higher variability of breathing rate^21–24^, whereas ventilatory response is reduced, suggesting a changed metabolic control of respiratory drive during REM compared to NREM sleep^23,25^. Moreover, REM sleep is often accompanied by low muscle tone, known as REM atonia^9^. REM atonia affects some skeletal muscles, including upper-airway muscles and limbs^9,21^, which increases breathing efforts and decreases limb movement. These breathing efforts and body movements produce sounds.

During sleep, there is an elevated resistance in the upper airway, which is reflected by amplification of air-pressure oscillations during breathing. These air-pressure oscillations are perceived as breathing and snoring sounds and carry information regarding the airway temporal structure and path^26^. Body movement usually involves friction with bedding fabrics, which has a distinct sound signature. These breathing and movement sounds can be exploited to evaluate MSS.

The use of audio analysis to evaluate sleep addresses some of the PSG limitations, making the sleep diagnosis procedure simpler, with higher comfortability to enable maintaining natural sleep. Recently, we have shown that it is possible to detect breathing sounds accurately^24,27,28^, and to distinguish between sleep and wake states using breathing sounds analysis^29,30^, and even presented a proof of concept for detection of REM on a small cohort^31^. Similar findings regarding the differences between snore properties of REM and NREM were also identified in more recent studies^28,32^. In this study, for the first time, a whole night non-contact audio-based MSS evaluation system is proposed; this system was designed and evaluated on a large subject database. By using a large dataset, we were able to add temporal information of acoustic properties throughout sleeping time rather than combining two separate models for acoustic properties and sleep patterns^31^.

We hypothesize that MSS activity can be estimated using audio signal analysis of sleeping sounds including breathing, movement, and even environmental sounds that might awaken the sleeper. The objectives of our study are: 1) to develop a sleeping sound analysis (SSA) algorithm for distinguishing wake, REM-sleep, and NREM-sleep using non-contact technology; 2) to reliably estimate sleep quality parameters such as total sleep time, sleep latency, sleep efficiency, wake after sleep onset time, REM latency, REM percentage, NREM percentage, and the number of REM cycles; and 3) to validate the proposed approach in comparison to PSG.

## Results

### Subjects and sleep quality parameters

Two hundred and fifty subjects referred to PSG evaluation were included in this study (Supplementary Table S1). Supplementary Fig. S1 illustrates individual big-data visualization for the design study (*n* = 150) and validation study (*n* = 100). All included subjects are typical to our region (Israel) and represent a vast range of age, body mass index (kg × m^−2^) (BMI), and apnea-hypopnea index (events × hr^−1^) (AHI). Data were split according to the following rule: all subjects with double recording (see Methods, “Audio recordings”) participated in the design study. The validation dataset consisted of *n* = 100 subjects (see Methods, “Data and statistical analyses”).

No significant differences in subject anthropometric parameters or AHI were found between system design and validation study groups (Supplementary Table S1). Both design and validation groups have, on average, moderate obstructive sleep apnea (OSA) that is more severe during REM vs. NREM sleep. Audio estimation of AHI (AHI_EST_) for the validation dataset was 11.8 ± 15.6, 18.6 ± 23, and 10.6 ± 23 events × hr^−1^ for estimated total sleep, REM, and NREM, respectively (see below); Concordance correlation and absolute error between AHI_PSG_ and AHI_EST_ was 0.90 and 4.7 ± 3.9 events × hr^−1^, respectively.

### Macro sleep stages estimation

**Figure 1:**
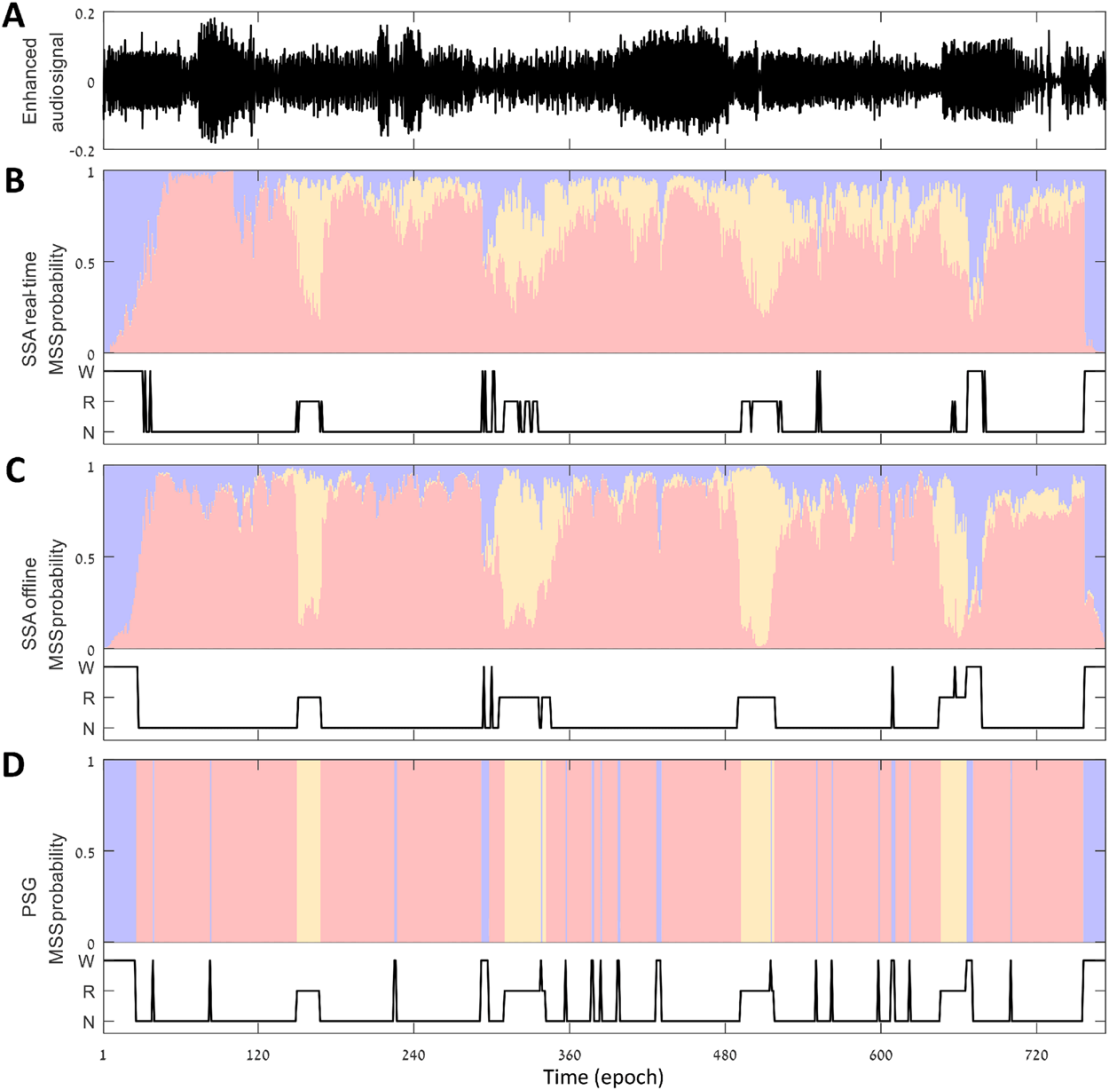
Whole night sleep sound analysis estimation. (**A**) Enhanced audio signal by adaptive noise reduction algorithm. (**B**) Real-time probability score of MSS. (**C**) Offline probability score of MSS. (**D**) MSS estimation based on PSG. MSS estimations are presented as likelihood probability score. MSS – macro sleep stage, SSA – sleep sound analysis, PSG – polysomnography (PSG), W – wake (blue), R – rapid eye movement sleep (orange), and N – non- rapid eye movement sleep (red). Data was collected from an 18-year-old female, BMI 32, apnea-hypopnea index 2. This subject exhibits epoch-by-epoch agreement with PSG of (88%, 0.66) and (92%, 0.80) for real-time and offline estimation, respectively (accuracy, kappa).

Figure 1 illustrates an example of a whole night (6:26 h) SSA of a subject from the validation dataset. Enhanced audio signal of the raw audio was achieved by using an adaptive noise reduction algorithm. Estimation of probability scores of MSS was calculated using a real-time analysis followed by offline MSS estimation analysis (Fig. 1B,C). The real-time analysis uses the information that was acquired up to the time the current epoch is being processed (see Methods section). Offline analysis was applied following completion of whole night data acquisition and obtaining the real-time MSS estimation. This offline procedure recalculates the real-time probability scores by utilizing futuristic probability scores, and hence increasing credibility/confidence scores regarding the decision of MSS classification (wake, REM, and NREM). A 92% agreement (epoch-by-epoch, 30 sec), and Cohen’s kappa coefficient of 0.80 was found in this subject between SSA estimation and PSG (Fig. 1C,D).

For real-time MSS classification, system performances were analyzed for each subject using the validation dataset (*n* = 100) (Table 1). The overall accuracy between PSG and SSA per subject was 82.2 ± 6.4%, with Cohen’s kappa coefficient of 0.59 ± 0.12. When simplifying the MSS estimation into a two-class decision of sleep or wake, overall accuracy of 90.3 ± 5.3% and Cohen’s kappa coefficient of 0.62 ± 0.15 was found. Table 2 summarizes a breakdown (confusion matrix) of system accuracy between SSA and PSG for each sleeping stage. For offline MSS classification, a very good estimation agreement between PSG and SSA was found, showing an accuracy of 86.9 ± 4.8%, with Cohen’s kappa coefficient of 0.69 ± 0.11 per subject. When simplifying the MSS estimation into a two-class decision of sleep or wake, an overall accuracy of 91.7 ± 4.3% and Cohen’s kappa coefficient of 0.68 ± 0.14 was found.

**Table 1:** System performance – agreement between SSA and PSG.

Classifications		Accuracy		Cohen’s kappa	
		Mean ± SD	Median (95% CI)	Mean ± SD	Median (95% CI)
Real-time	WRN (3-Class)	82.2 ± 6.4	82.6 (69.0–93.3)	0.590 ± 0.122	0.598 (0.323–0.798)
	WS (2-Class)	90.3 ± 5.3	90.9 (75.0–98.4)	0.619 ± 0.147	0.642 (0.296–0.890)
Offline	WRN (3-Class)	86.9 ± 4.8	87.3 (76.3–95.0)	0.694 ± 0.113	0.700 (0.377–0.869)
	WS (2-Class)	91.7 ± 4.3	92.7 (81.1–98.0)	0.676 ± 0.145	0.704 (0.341–0.922)

System performance was evaluated on the validation dataset using the real-time and offline estimations protocols. Accuracy and Cohen’s kappa coefficient were calculated between sleep sound analysis (SSA) and polysomnography (PSG) for each subject using epoch-by-epoch (30 sec) analysis. Data was analyzed for three classes of wake (W), rapid-eye-movement (R), non-rapid-eye-movement (N), and two classes of wake (W) vs. sleep (S). Values are presented as a mean percent agreement ± standard deviation (SD) and median 95% confidence interval (95% CI).

**Table 2:** System performance – confusion matrix of macro sleep stages.

		SSA Real-time			SSA Offline		
		**W**	**R**	**N**	**W**	**R**	**N**
**PSG**	**W**	**11744**	314	3642	**12403**	141	3156
		**(74**.**8%)**	(2.0%)	(23.2%)	**(79**.**0%)**	(0.9%)	(20.1%)
	**R**	428	**5631**	3248	297	**6738**	2290
		(4.6%)	**(60**.**5%)**	(34.9%)	(3.0%)	**(72**.**4%)**	(24.6%)
	**N**	4393	2909	**52066**	3562	1484	**54322**
		(7.4%)	(4.9%)	**(87**.**7%)**	(6.0%)	(2.5%)	**(91**.**5%)**

System performance was evaluated on the validation dataset using the real-time and offline estimations protocols. Confusion matrix of agreement between the PSG and SSA in identifying three macro sleep stages (W – wake, R – rapid-eye-movement, N – non-rapid-eye-movement). Data in is presented as epochs (percentage) from the entire validation dataset.

Supplementary Fig. S2, shows the detection (precision and recall) of each MSS (wake, REM, and NREM) among subjects for both realtime and offline estimation. Supplementary Fig. S3, shows a performance breakdown of given epoch estimation (wake, REM, and NREM) into subject characteristics.

### Sleep quality assessment

Using the detected MSS from the offline analysis, sleep quality parameters were calculated (Table 3). Comparison between SSA parameters and PSG sleep parameters revealed very good agreements and no consistent difference was found. The absolute error (magnitude of differences), was found to be minimal in all sleep quality parameters (TST: 11.7 ± 11.5 min, SL: 6.8 ± 13.8 min, WASO: 14.8 ± 17.7 min, RL: 27.6 ± 57.5 min, SE: 2.6 ± 2.3%, RP: 3.5 ± 3.8%, NP: 4.1 ± 4.2%, and RC: 0.4 ± 0.7).

**Table 3:** Sleep Quality Parameterization.

Parameter	PSG	SSA	Difference (SSA-PSG)	Absolute difference
TST (min)	343.4 ± 51.2	346.7 ± 49.55	3.3 ± 16.1	11.7 ± 11.5
	(220.5–419.5)	(236.0–426.0)	(−36.0–33.0)	(0.0–44.5)
SL (min)	30.2 ± 27.7	26.6 ± 21.0	−3.6 ± 15.0	6.8 ± 13.8
	(0.5–116.5)	(3.0–99.5)	(−18.5–24.0)	(0.0–24.5)
SE (%)	89.1 ± 7.9	88.7 ± 9.2	−0.5 ± 5.2	3.5 ± 3.8
	(70.1–99.0)	(64.4–99.4)	(−15.7–9.1)	(0.0–15.7)
WASO (min)	41.7 ± 30.9	44.4 ± 36.0	2.6 ± 23.0	14.8 ± 17.7
	(4.5–121.5)	(3.0–146.0)	(−35.5–68.5)	(0.0–68.5)
RP (%)	12.1 ± 7.1	13.7 ± 7.3	1.5 ± 3.1	2.6 ± 2.3
	(0.0–28.0)	(0.7–30.7)	(−3.7–8.4)	(0–8.4)
NP (%)	76.9 ± 8.6	74.9 ± 9.3	−2.1 ± 5.5	4.1 ± 4.2
	(55.4–93.5)	(52.9–89.3)	(−15.1–5.7)	(0.1–15.1)
RL (min)	192.1 ± 90.2	173.8 ± 82.4	−20.4 ± 60.4	27.6 ± 57.5
	(69.5–383.5)	(63.9–374.5)	(−199.7–32.7)	(0.0–199.7)
RC (#)	2.0 ± 1.1	2.2 ± 1.0	0.2 ± 0.8	0.4 ± 0.7
	(0–4)	(0–4)	(−1–3)	(0–3)

Data were calculated from the validation dataset (*n* = 100). Difference – was calculated between sleep sound analysis (SSA) and polysomnography (PSG) for each subject; Absolute difference – quantified the overall magnitude of differences among measurements; TST – total sleep time; SL – Sleep latency; SE – Sleep efficiency; WASO – Wake time after sleep onset; RP – Rapid-eye-movement percentage; NP – non-Rapid-eye-movement percentage; RL – REM latency; RC – number of REM cycles; Values are presented as mean ± SD (95% CI) between subjects.

**Figure 2:**
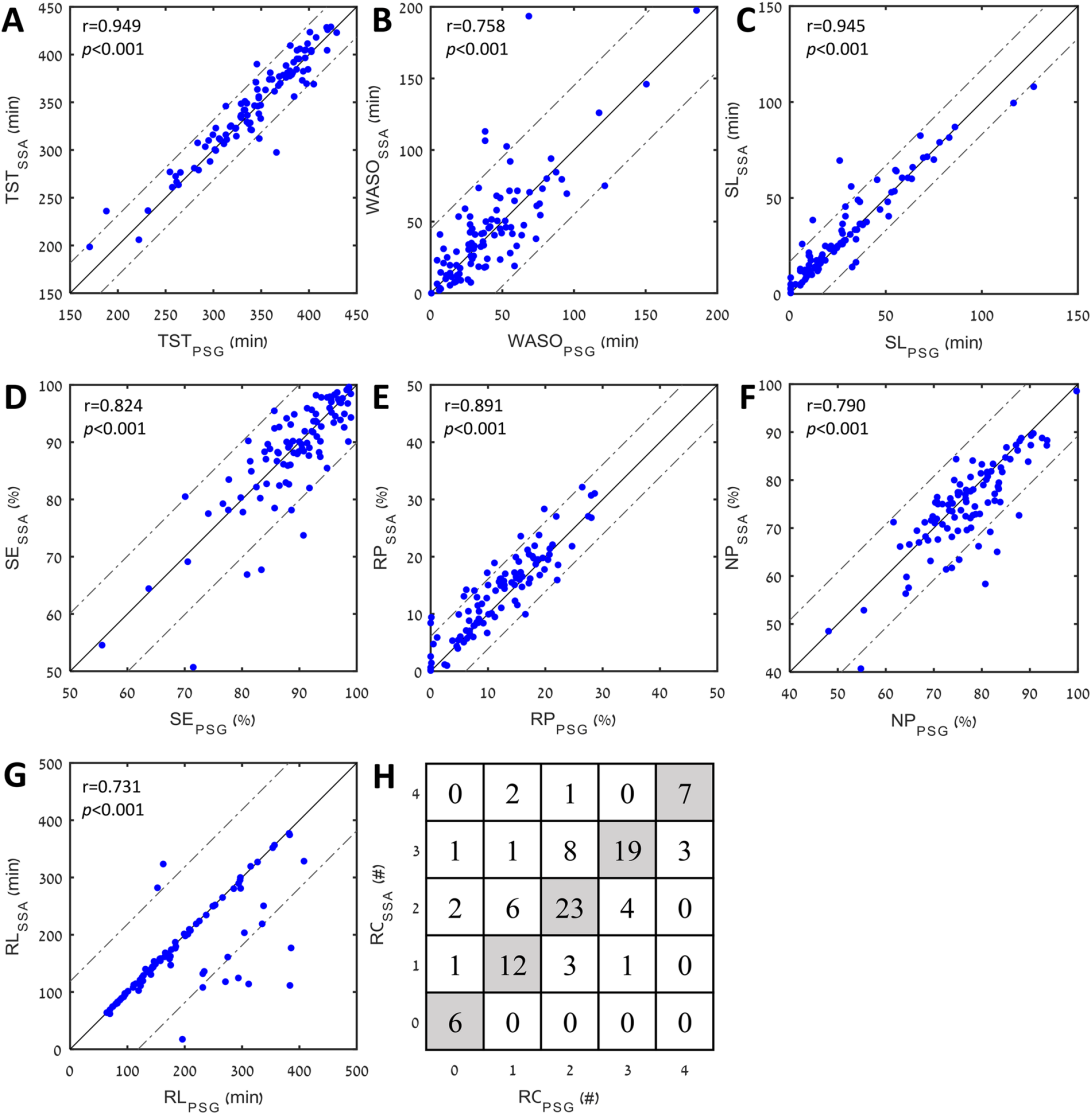
Comparison of sleep quality parameters between SSA and PSG. Data was taken from the validation dataset (*n* = 100), each point represents a subject. (**A**) TST – Total sleep time (minutes); (**B**) WASO – Wake after sleep onset (minutes); (**C**) SL – Sleep latency (minutes); (**D**) SE – Sleep efficiency (percentage of total sleep time); (**E**) RP – Rapid-eye-movement sleep (percentage of total sleep time); (**F**) NP – Non-rapid eye movement sleep (percentage of total sleep time); (**G**) RL – rapid eye movement latency (minutes); (**H**) RC – number (#) of rapid eye movement cycles, presented as confusion matrix. Solid line – line of identity between polysomnography (PSG) and sleep sound analysis (SSA); dashed lines – 95% confidence interval; r – concordance correlation coefficient.

Figure 2 shows evaluation of these sleep quality parameters using the validation dataset. The individual data points of subjects in the validation group grossly converge with the line of identity (solid black line) in all sleep quality parameters. Concordance correlation coefficient revealed that sleep quality parameters according to PSG correlate with all SSA sleep quality parameters (Fig. 2A–H). Moderate agreement between SSA and PSG estimation of the number of REM cycles was found (Cohen’s kappa coefficient = 0.56). Please note that REM latency (RL), in Fig. 2G, shows an almost perfect match for most subjects. Interestingly, those who did not match have a diversion of about 100 min, indicating either missing an entire REM cycle or detecting a REM cycle when it is absent from the PSG.

Examining the Bland-Altman plots^33^ and comparing the proposed SSA approach versus PSG, we found good agreement. The plots of all sleep quality parameters show no significant, consistent bias (Supplementary Fig. S4); this implies reliability of the estimated parameters.

**Figure 3:**
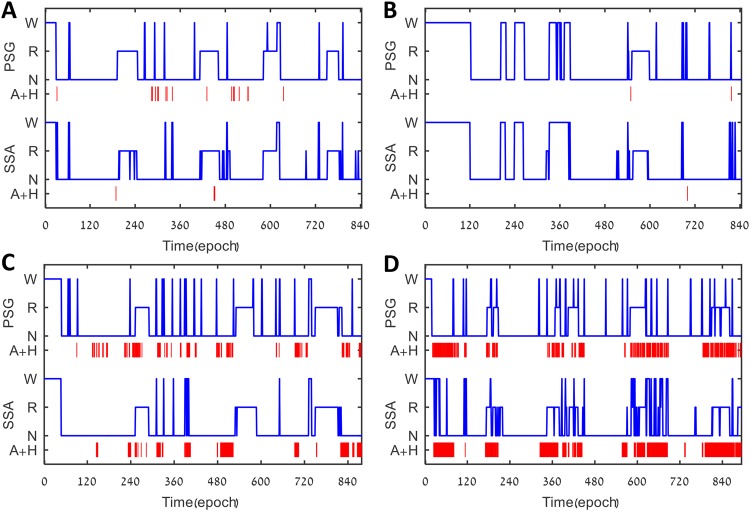
Comparison of MSS estimation and detection of AHI between PSG and SSA. (**A**) Healthy subject (AHI < 5); (**B**) Subject with long R latency (440 epochs); (**C**) Subject with moderate sleep apnea (AHI = 18), obstructive events appear in both R and N sleep; (**D**) Example of R-related sleep apnea (AHI 55 and 21 in R and N sleep, respectively). MSS – macro sleep stages; PSG – polysomnography; SSA – sleep sound analysis; R – rapid-eye-movement sleep; N – non-rapid-eye-movement sleep; AHI – apnea-hypopnea index (events/hr) (A + H).

The proposed SSA system shows good agreement for detection of sleep disorders for individual subjects. Figure 3 shows examples of four cases illustrating agreements in the number of RCs determined by PSG versus SSA. MSS estimated by SSA was robust in detecting subjects with “first night effect” (Fig. 3B), a condition that manifests prolonged RL compared to subjects without this effect (Fig. 3A). MSS estimation according to SSA was robust in the detection of REM and NREM epochs and was not affected by the presence of OSA (Table 3). The absolute error between AHI_PSG_ *vs*. AHI_EST_ was 10.1 ± 10.4 and 4.5 ± 4.0 events × hr^−1^ for REM and NREM, respectively. Finally, combining SSA estimation of MSS and audio estimation of apneic events revealed that this integrated system could accurately detect NREM/REM-related OSA (Fig. 3C,D).

## Discussion

Chronic disorders of sleep are a global problem that can adversely affect daily functioning, safety, quality of life and health^3,4^. Sleep staging and sleep architecture are essential for the understanding of sleep pathology and its effect on health. The current standard for PSG sleep staging is visual data review by certified sleep technicians following standardized rules^13^ for scoring S1, S2, slow-wave sleep (SWS), REM, and wake, but for this study scores were grouped into macro sleep stages (MSS), i.e., wake, REM/non-REM (NREM). Sleeping in a laboratory setting is unusual for subjects and can, therefore, result in altered sleep architecture, such as present in the well-known first night effect^34^, and it is likely that disorders are missed if only one to two nights can be assessed. Hence, there is tremendous need to provide user-friendly, accurate, and noninvasive sleep-wake monitoring^5,18^.

In this paper, a novel method for evaluation of MSS patterns using a non-contact microphone is proposed. This method utilizes analysis of sleeping sounds generated by the sleeper and machine-learning techniques to automatically estimate MSS. The proposed system is capable of estimating MSS in both real-time (online) and offline manners, where real-time estimation can be useful for sleep-intervention applications such as a smart alarm clock, and offline for the generation of sleep quality reports.

Performance of our audio-based systems are encouraging, yielding an average agreement of 82% (κ = 0.59) and 87% (κ = 0.69) for MSS estimation using online and offline estimations, respectively, and 90% (κ = 0.62) and 92% (κ = 0.68) for binary sleep-wake decision. Using the offline estimated epoch labels (wake/REM/NREM), we calculated eight acceptable sleep quality parameters. The average error of total sleep time was 11.7 min, sleep latency 6.8 min, sleep efficiency 3.5%, wake after sleep onset 14.8 min, REM percentage 2.6%, NREM percentage 4.1%, REM latency 27.6 min, and REM cycles was 0.4 cycles. In most cases (84%), REM latency was accurately detected (3.0 min error on average). In other cases (16%), the relatively large latency error was the result of a misdetection of the first REM cycle or a false detection of REM when it was absent.

### The sound of sleep

The main idea beyond this approach is that during sleep, the sleeper generates two kinds of sound sources – vocal sounds (such as respiration, murmurs, talking, and coughing), and sounds due to movement in bed. In previous work^29^ we showed that sleep and wake activity is closely associated with breathing sounds and breathing properties, as the central controls of ventilation and upper airway patency are tightly connected^35^. The upper airway is a complex structure comprising several pharyngeal muscles that are essential for several tasks including speech and breathing^36^. While sleeping, pharyngeal muscle activity decreases, causing narrowing of the airway’s patency and increasing its resistance^35^, this in turn, alters the generated sound properties, frequently perceived as louder breathing or snores.

Moreover, REM is an intermediate state between deep sleep and wakefulness, where the body is sleeping while mind activity is high, also known as paradoxical sleep. This sleeping state is expressed with muscle atonia (paralysis of large muscles), which is the hallmark of REM in contrast to other vigilance states^9^, and the elevated brain activity is expressed in varying ventilation demands^22,25^. Additional information regarding sleep-wake activity can be found in body movements, which are commonly used in actigraphy-based technologies^37^. The assumption in these technologies is that sleep is associated with lack of body movements whereas wakefulness is associated with elevated activity; this assumption is valid most of the time but not entirely, since the sleeper can be turning his body during sleep or even staying still while trying to fall asleep. Both body movements and breathing efforts generate sounds (sleep sounds) and can be acquired and quantified using a non-contact microphone. Using audio processing algorithms, the actual MSS can be reliably estimated by estimating airways resistance and other physiological changes that take place during sleep. Supplementary Fig. S5 – “sound of sleep” shows the sound intensity distribution of the sleeping sound content.

### Sounds analysis

Extracting MSS activity pattern from sleeping sounds using a non-contact microphone is challenging. Since sleeping sounds may be contaminated by background noises, it is essential to improve SNR prior to analysis in order to detect and quantify breathing and body movement properties. Analyzing our acquired audio signals, we measured the room (Soroka settings) background noise level in the room to be 37 ± 2 dB SPL. To achieve a proper signal enhancement, we used an adaptive spectral subtraction technique^27^ that subtracts the estimated changing background noise at a 30-sec epoch resolution. In previous works^24,27,29,31^ we showed that this step is beneficial for detecting low-intensity breathing sounds. By applying this technique, background noise was considerably reduced, similar to earlier studies^27,38^. Once the audio signal is enhanced, breathing and body movement detectors can be reliably applied. We used our high performance (>98% detection accuracy rate) snore detector^27^ module. Supplementary Fig. S6 shows the body-movement and breathing detection performances as a function of the measured SNR. In an earlier study^24^, we compared breathing rate estimated using RIP and using audio analysis; results showed a mean average error of 0.42 ± 0.17 BR/min and Pearson correlation of 0.68 ± 0.16 per subject, with an overall correlation of 0.97 for comparing average breathing rate between subjects.

**Figure 4:**
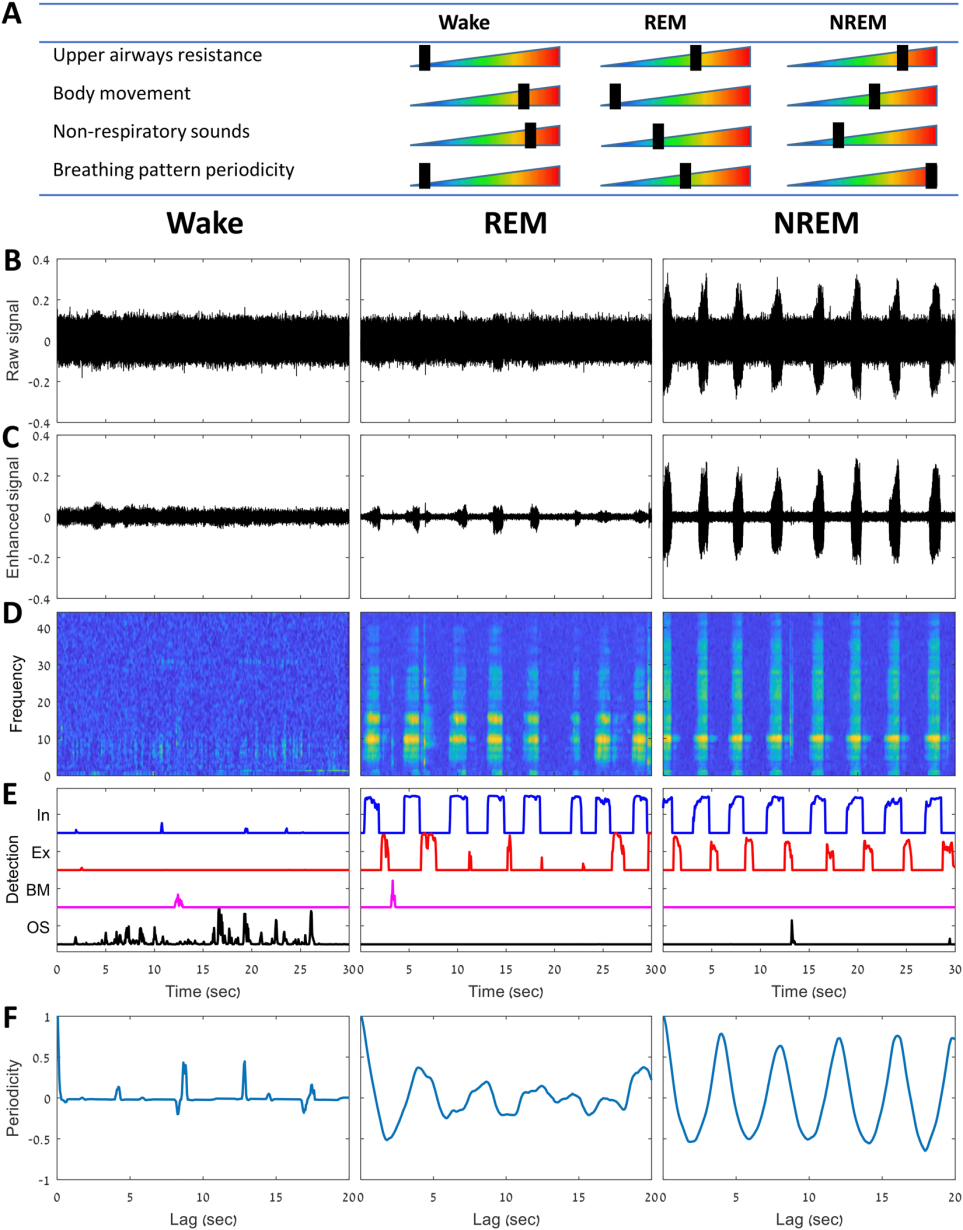
Macro sleep stages estimation. (**A**) Summary of guidelines used for developing MSS estimation system. Alterations in sleep stages across sleep are associated with physiological changes such as: upper airways resistance, breathing pattern periodicity, body movements, and non-respiratory sounds (coughing, itching, murmurs). All these physiological changes have recognizable sound patterns that could be detected and analyzed. Black rectangle marks the relative effect of each parameter using color gradient; weak effect is illustrated by cold colors (left side of the triangle); strong effect is represented by warm colors (right side of the triangle). For example, body movement is absent in REM, high during wake, and lower during NREM. (**B**) Raw audio signal; (**C**) Enhanced audio signal following noise suppression, (**D**) spectrogram, warmer colors represent higher sound intensity (dB); (**E**) Detection of inhalation (In, blue color), exhalation (Ex, red color), body-movement (BM, pink color), and other sounds (OS, black color) values are expressed as a likelihood score. Higher values (upright) indicate higher likelihood score; (**F**) periodicity measurement calculated as autocorrelation function of the detected breathing curves in E. Note the periodic pattern in NREM that decreases in REM and is almost absent in wake. MSS **–** macro sleep stages, i.e., wake, rapid-eye-movement (REM), and non-rapid-eye-movement (NREM) sleep. Data show one representative epoch (30 sec) during wake, REM, and NREM, from 53-year-old male. In – inhalation; Ex – exhalation; BM – body-movement; OS – other sounds.

We developed a set of features that are specifically designed to distinguish between the three MSS phases (Supplementary Table S2, Fig. 4). These features can be classified into five categories: *within-breathing*, *between-breathing*, *body-movement*, *background noises*, and *personalization*.

The *within-breathing* features are designed to capture and quantify breathing characteristics for every epoch. The basic idea here is that these features are able to detect the influence of the pharyngeal muscles’ activity on the upper airway and on the sound generation itself as it is determined by the upper airway anatomy. These changes in muscle activity are expressed as increased/decreased breathing sound intensity, inhale/exhale durations, spectral contents, entropy and more.

The *between-breathing* features are designed to quantify the relations between several breathing cycles for every epoch – to form the breathing pattern. It was well-established that changes in vigilance states strongly affect breathing rate and regularity in humans and animals^22,25,35^.

The *body-movement* features are designed to detect and quantify body movement sound events generated by the subject moving in bed. The basic idea here is similar to the actigraphy approaches, which assume that sleep is associated with lack of movement and vice versa. Moreover, during REM sleep, body movement should be absent due to a mechanism that is supposed to paralyze our limbs in order to prevent us from harming ourselves during dreams^9^.

The *background noises* features are designed to assess noises that are either generated by the subject (such as talking, sneezing, coughing) or by a third party (such as dog barks, slamming doors, cars) and might awaken the subject. It is presumable that the louder (and non-constant intensities) could awaken the sleeper.

The last set of features is the *personalization* features, which aim to tune (calibrate) the analysis to the subject’s anthropometric parameters and to add chronicity to the estimation. We hypothesize that useful information is located in the time index of the examined epoch as it may express in a typical sleep pattern, e.g., the first epochs are most likely to be “wake” followed by roughly 90-minute sleep cycles throughout the night. This finding was previously proved^31^ in the Markov model trained over sleep patterns. Future studies are need to validate our approach on narcolepsy or insomnia patents. Features importance assessment is provided in Supplementary Table S2 and visualized in Supplementary Fig. S7.

Sleep architecture usually involves sleep/wake and REM/NREM patterns. To exploit these temporal patterns, we constructed two time-series classifiers, one for real-time estimation, and one for offline estimation. The real-time classifier utilizes the knowledge acquired from the acoustic features of the previous epochs and the current epoch; the offline classifier utilizes the entire (whole-night) knowledge to estimate more reliable sleep patterns.

### Comparison to existing approaches

Limited information is available about sleep evaluation using non-contact technologies, especially using audio-based approach. To the best of our knowledge, our study is the first to show that non-contact microphones can reliably estimate MSS. Table 4 shows a comparison between our audio-based system and existing sleep trackers that use minimal sensors to estimate the two-class decision of sleep/wake and the three-class decision of wake/REM/NREM.

**Table 4:** Comparison to other sleep monitor devices.

Approaches			Sleep estimation accuracy (kappa)		Sleep parameters correlation (absolute error)								Validation	
Research/product	Sensor used	Class	Wake Sleep	Wake REM NREM	TST	SL	SE	WASO	RP	NP	RL	RC	Cohort	Reference
**This study (SSA)**	non-contact microphone	3	91.7% (0.68)	86.9% (0.69)	0.95 (11.7)	0.95 (6.8)	0.82 (3.5)	0.76 (14.8)	0.89 (2.6)	0.79 (4.1)	0.73 (27.6)	0.7 (0.4)	100	PSG
DeepSleepNet^19^	EEG	5	97.3% (0.86)	93.8% (0.86)	—	—	—	—	—	—	—	—	62*	PSG
Zhu *et al*.^39^	EEG	5	97.7% (0.95)	92.8% (0.88)	—	—	(7)	—	—	—	—	—	8	PSG
Zeo^40^	EEG	4	92.6% (0.65)	85.4% (0.69)	0.95	0.42	0.95	0.9	0.51	—	0.02	—	26	PSG
WatchPAT^41–43^	accelerometer oximeter PAT Snore	4	86.3% (0.56)	77.2% (0.57)	0.66–0.68	—	—	—	0.58	—	—	0.34	31	PSG
Earlysense^44^	Mattress pressure sensor	4	90.5% (0.68)	77.8% (0.56)	0.87	—	—	—	—	—	—	—	85	PSG
PulseOn^45^ (SmartWatch)	PPG, accelerometer	3	–	#81.4%	–	–	–	–	–	–	–	–	108	PSG
Tararaidze *et al*.^46^	RIP	3	88.4% (0.67)	80.4% (0.65)	—	—	—	—	—	—	—	—	29	PSG
Tararaidze *et al*^47^.	Bio-motion sensor (RF)	4	86.3% (0.57)	75.9% (0.55)	—	—	—	—	—	—	—	—	32	PSG
Zaffaroni *et al*.^48^	Bio-motion sensor (RF)	4	90.6% (0.51)	79.2% (0.53)	–	0.41 (8.0)	0.67 (4.8)	—	—	—	—	—	20	PSG
Philip *et al*.^49^	Bio-motion sensor (RF)	2	78% (0.38)	—	(50)	—	(12)	—	—	—	—	—	113	PSG
Sleep Hunter^50^	Smartphone’s microphone lumination accelerometer	3	†	#73% (0.44)	—	†	†	†	—	—	—	—	45	Zeo
Actigraph^52^	accelerometer	2	86.3% (0.36)	—	—	—	—	0.6	—	—	—	—	77	PSG

TST – total sleep time; SL – Sleep latency; SE – Sleep efficiency; WASO – Wake time after sleep onset; RP – Rapid-eye-movement percentage; NP – non-Rapid-eye-movement percentage; RL – REM latency; RC – number of REM cycles; SSA – Sleeping sound analysis; EEG – electroencephalogram; PPG-photoplethysmography; RIP – respiratory inductance plethysmography; PSG – Polysomnography. The approaches class column represents the maximum classes seperatable using the respective approach. Underline mark “—” indicates that this performance score was not originally included in the paper and was estimated by us using the complementary data available in the paper. † indicates that the wresearches considered Wake as REM; * indicates 31-fold cross-validations over 62 subjects; # indicates two-class, REM vs. NREM classification, in this case, our SSA yeilds 93% (0.76). It is important to mention that our sleep parameters correlations were calculated using concordance-correlation while other approaches did not report and might have used Pearson correlation values instead.

From Table 4 one can see that there are contact-based technologies such as EEG^39,40^ and WatchPat^41–43^, minimal contact devices such as EarlySense^44^, smartwatch^45^, and respiratory belts^46^, and non-contact devices that use radio frequency (RF) to detect a subject’s movement^47–49^. Lately, there are smart-watches and apps for smartphones^50^. However, most of these technologies were not clinically validated, nor reported for accuracy. Smartwatch and actigraphy-based sensors rely somewhat on body movement estimations and offer relatively high comfort. However, it is generally recognized that sleep evaluation using body-movement-based technologies (contact and non-contact) is biased^51,52^, since any lack of movement is interpreted as sleep while movement is interpreted as awake. Consequently, some of the advanced smartwatches use additional information, such as heart rate, that helps estimating the sleep stages^53^. In addition to body movement, our approach of estimating MSS uses the additional information presented in breathing sounds. In contrast to the existing movement-based technologies, we found that our system performance is less influenced by any physiological variables, such as age, BMI, AHI, and gender (see Fig. S2). Overall, it seems that our audio-based approach may provide a simple, convenient, and easy to use technology for estimating macro sleep stages.

### Strengths and limitations

We provide evidence that MSS activity can be reliably estimated remotely by audio analysis of sleeping sounds. In this study 299,437 epochs were analyzed from 250 subjects who were referred to sleep evaluation; these subjects represent a diverse population, across a wide range of age, BMI, and AHI, and from both genders, but are not strictly a generalizable population. Our proposed non-contact technology may enable more natural sleep that is not affected by the equipment. It is expected that the wear and tear and costs will be relatively low; however, further studies should determine whether this technology is valid in at-home conditions and is cost-effective. In Israel, the transition of this technology to at-home sleep evaluation mainly depends on health maintenance organization reimbursements policy for at-home sleep study.

Perhaps one of the short-comings of this study is the neglect of differentiation between S2 and SWS, as it is standard in PSG recordings, clinical evaluation, as well as recommended by the AASM manual^13^. Additionally, our proposed method cannot separate S1 sleep from NREM. Currently, our proposed system may be suitable for OSA diagnosis. Further studies are needed to investigate whether audio analysis can separate NREM into light and deep sleep stages (S1–S4) and to validate our system in detecting other sleep disorders such as REM behavior disorder or insomnia. It should be recognized that the main limitation of this technology is its sensitivity to low SNR, which results from microphone’s quality or due to quiet sleeping sounds relative to high environmental background noise. Our performance evaluations show a decent correlation between SNR and system performances. In post-analysis (after the system evaluation was made), we investigated the low-SNR epochs that were misclassified and we found that many epochs were classified as “wake”. We hypothesized that this decision was made since wakefulness is usually characterized with very quiet breathing sounds (translated in very low SNR), and sometimes without body movements. The same conditions were observed when the patient left the room for the toilet. Also, at the current state of operation, our system is limited to operating in a single-subject environment (one person in the room), since another person’s sleeping sounds can interrupt. Further studies should investigate methods to cope with several subjects in the room and to further improve SNR mechanically and algorithmically.

### Summary

One of the main goals of sleep medicine today is to improve early diagnosis and treatment of the “flood” of subjects presenting with sleep disorders. New simple technologies are needed to improve patient accessibility to sleep diagnosis; this will reduce the cost of management and treatment and improve quality of life and health. This study presents a pioneering approach for determining MSS pattern using non-contact audio-based signals. We found that by analyzing sleeping sounds in both real-time and offline procedures, a reliable estimation of sleeping states and sleep quality parameters can be produced. This study highlights the potential of sleeping sound analysis to measure sleep in research and clinical situations in great comfort.

## Methods

### Developing the concept of macro sleep stages estimation

Changes in MSS from either NREM, REM, or wakefulness are associated with alterations in breathing pattern periodicity^29^, upper airway muscle tone (contributing to pharyngeal resistance)^35^, and changes in body movements^54^. All these activities are accompanied with recognizable sound signatures that could be detected and analyzed. The following concept was used to develop the classification of wake, REM, and NREM (Fig. 4A). During sleep (in contrast to wakefulness) there is an increase of upper airway resistance due to decreased activity of the pharyngeal dilator muscles, which is reflected by amplification of air-pressure oscillations during breathing. These air-pressure oscillations are perceived as breathing sounds during sleep^27,28^. In addition, REM, NREM, and wakefulness are associated with lack of, some, and considerable body movement, respectively^55,56^. Breathing pattern is more periodic and consistent in deep NREM sleep compared to REM and wakefulness^22,25,29,35^. It is possible to detect (and distinguish) whole night respiratory and non-respiratory sounds (such as changes in sleeping position, coughing, talking, bedding noise) even below the environmental background noises^27^. Fig. 4B–F illustrates typical data during wake, REM, and NREM from one subject’s design dataset. Notice how control of ventilation is manifested by periodic breathing pattern during NREM, which degrades in REM and is almost absent in wakefulness (Fig. 4E,F). Accordingly, the relative effect of each physiological parameter was taken into consideration in developing the acoustic features (see below).

### Setting

The research was conducted in the university-affiliated sleep-wake disorder center and in the biomedical signal processing research laboratory, Be’er-Sheva, Israel. The Institutional Review Committee of Soroka University Medical Center approved this study protocol (protocol number 10141) and all methods were performed in accordance with the relevant guidelines and regulations. The institutional review board waived the need for written informed consent from the participants.

### Sleep study and subjects

During 2008–2016, 250 subjects reported to the laboratory at 20:30 and were discharged at 06:00 the following morning. They were encouraged to maintain their usual daily routine and to avoid any caffeine and alcohol intake on the day of the study. The laboratory environment was sleep-friendly according to recommendations of the National Sleep Foundation^57^. PSG (SomniPro 19 PSG, Deymed Diagnostic, Hronov, Czech Republic) scoring including wake, rapid-eye-movement (REM), and non-REM (NREM) pattern was determined by a trained technician and underwent a second scoring by one of the investigators (AT) to reduce human scoring mistakes; scoring followed the American Academy of Sleep Medicine criteria^13^. The scoring included labeling of each (30 sec) epoch as wake, REM-sleep, or NREM-sleep using the PSG signals. In order to train and validate our system, we split our sleep data into design dataset (*n* = 150) and validation dataset (*n* = 100); see Supplementary Table S1.

### Audio recordings

A digital audio recording device (Edirol R-4 pro, Bellingham, WA, USA) with a directional microphone (RØDE, NTG-1, Silverwater, NSW, Australia) was placed at a distance of one meter above the subject’s head and used for acquiring the audio signals. An additional handy audio recording device (Olympus LS-5) was placed horizontally on a dresser beside subject’s head and was used for all of the subjects from the design dataset to achieve a system that is reliable and robust to other recording specifications and even different distance or angle. We treated the additional records as new subjects in the design dataset. The audio signals were stored along with the PSG signals for later analysis. Each audio signal was synchronized with the PSG study at 10 ms resolution according to cross-correlation technique between the PSG snore intensity level channel and the digital audio signal (after matching the energy sampling rate). To simulate real-time analysis, audio signals were fed into the validation system as a stream.

### The MSS pattern estimation algorithm

Supplementary Fig. S8 shows the block diagram of the proposed sleep evaluation algorithm for the design and validation phases of the study. The algorithm’s input is a stream of audio signal at 44.1 kHz (16 bit per sample); the process is composed of four essential steps: A) pre-processing and noise reduction, B) breathing and body movement detection, C) feature extraction, D) MSS classification. The output of this algorithm is MSS estimation at a 30-sec epoch resolution. At the end of the recording, whole-night sleep quality parameters are calculated.

### Preprocessing & noise reduction

This step is applied to each audio signal (i.e., subject) in either design or validation phases.

In order to simulate a real-time analysis, the stored audio signal was streamed at 44.1 kHz in 50 ms packages (i.e., frame window of 2205 samples) for subsequent analysis.

These frames underwent adaptive noise suppression (spectral subtraction) process based on the Wiener-filter to remove background and stationary noises^27^. The use of the noise suppression in this system is crucial since it is designed to emphasize low-intensity breathing sounds by suppressing any stationary background noise, such as an air-conditioner or fan noises. This process relies on automatically and adaptively tracking background noise frames to estimate their spectra and subtracting them from the audio signal^27^. Any residual noises (transient noises) such as an analog clock or dripping faucet will survive this process; however, will be dealt with using the events detectors (see next section). Once the audio signal in each frame is enhanced, it was aggregated to form 30-sec epoch segments (i.e., concatenating 600 frames) matching the PSG scoring resolution. These 30-sec epoch segments present the building block for the MSS estimation in which features are extracted, and epoch state is calculated.

### Breathing and body movement detection

Two types of detectors were developed and applied: i) inhalation/exhalation detector and ii) body movement detector. The body movement detector structure was similar to the inhalation/exhalation detector^27^, using the same acoustic features, but with “body movement”/“non-body movement” annotated events for training. The outputs of these detectors are four probability curves (likelihood scores): for inhalation, for exhalation, for body movement, and for other sounds (non-movement and non-breathing sounds). These probability curves were calculated in a 50 ms resolution (frame rate), from each 30-sec epoch (see examples in Fig. 4E). Using these probability curves and the acoustic information in the epoch, MSS features are extracted.

### Feature extraction

Five sets of features (a total of 67 features) were developed and extracted from each epoch based on the audio content and the probability curves of respiration and body movement mentioned above. Features descriptions are presented in Table S2.

#### Within breathing features

consist of 33 features, which are designed to capture and quantify breathing characteristics for a given epoch. The hypothesis is that different vigilance states possess different upper-airways morphology due to the dilating muscles in the larynx^29^. During sleep, airways patency is higher than during wakefulness, hence breathing efforts become greater, which translates into several factors including louder breathing sounds, prolonged breathing duration, and different vocal sounds (snores). The entire “within breathing features” set is listed in Table S2A.

#### Between breathing features

consist of 12 features that are designed to quantify the relations between the breaths in a given epoch. The hypothesis is that control of ventilation is affected by different sleeping states^22,25^. These alternations in ventilation may affect fundamental respiration factors such as respiratory cycle period, respiratory duty cycle, and respiration consistency, and can be measured using sound analysis. These respiration factors are most likely to have more substantial variability during REM as opposed to NREM. The entire “between breathing features” set is listed in Table S2B.

#### Body movement features

consist of 10 features that are designed to quantify body movements. The hypothesis is that wakefulness is accompanied by relatively greater body movement, compared to NREM, while during REM sleep body movement should be absent by definition. There is a mechanism designated for paralyzing our limbs to prevent us from harming ourselves during dreams^9^. As a support for these hypotheses, many ambulatory sleep evaluation devices, including actigraphy, use this underlying assumption – that sleep is associated with lack of movement as opposed to wakefulness. The entire *Non-breathing features* set is listed in Table S2C.

#### Background noises features

consist of 8 features that are designed to assess noises that are either generated by the subject (such as talking, sneezing, coughing) or by a third party (such as dog barks, slamming doors, cars) and might awaken the subject. It is presumable that the louder (and non-constant intensities) could awaken the sleeper. The entire *Background noises features* set listed in Table S2D.

#### Personalization features

consist of 4 features that are designed to apply additional knowledge about subject physiology to the MSS decision. Three anthropometric parameters were included, age, gender, and BMI, as sleep quality is known to deteriorate with aging and/or obesity. Additional knowledge may come from the chronicity of the recording time (epoch’s index), where wakefulness is more probable during the first recording epochs. The entire *Personalization features* set is listed in Table S2E.

### MSS classification

Based ‘on the extracted features for each epoch (at a 30-sec resolution), and the temporal relation between adjacent epoch states, a time-series classifier was applied. A time series classifier can exploit the information needed for temporal MSS pattern estimation. In this work, we constructed a classifier for real-time MSS estimation (*C*^(*r*)^) based on the features information from the current epoch and the epochs before the tested epoch. For a complete sleep pattern estimation, we applied an additional time-series classifier on the estimated MSS scores (on the real-time estimations) that uses the information located ahead of the tested epoch, making MSS estimation more accurate (offline MSS classifier, *C*^(*o*)^). We trained our MSS classifiers on the design dataset with 80% of the subjects for training and 20% for development; see Supplementary Table S1.

#### Real-time MSS classification

The real-time classifier (*C*^(*r*)^) was composed of six sub-classifiers, adequate for six different periods. Each sub-classifier uses the information available for each epoch in the designated period.

$${C}^{(r)}({\bf{X}},t)=\{\begin{array}{ccc}{C}^{({r}_{1})}= 'Wake' & neurons=0 & \begin{array}{cc}{\rm{P}}{\rm{e}}{\rm{r}}{\rm{i}}{\rm{o}}{\rm{d}}\,1: & t\le 5\end{array}\\ {C}^{({r}_{2})}({\bf{X}},t) & neurons=100 & \begin{array}{cc}{\rm{P}}{\rm{e}}{\rm{r}}{\rm{i}}{\rm{o}}{\rm{d}}\,2: & 5 < t\le 20\end{array}\\ {C}^{({r}_{3})}({\bf{X}},t) & neurons=400 & \begin{array}{cc}{\rm{P}}{\rm{e}}{\rm{r}}{\rm{i}}{\rm{o}}{\rm{d}}\,3: & 20 < t\le 50\end{array}\\ {C}^{({r}_{4})}({\bf{X}},t) & neurons=1000 & \begin{array}{cc}{\rm{P}}{\rm{e}}{\rm{r}}{\rm{i}}{\rm{o}}{\rm{d}}\,4: & 50 < t\le 100\end{array}\\ {C}^{({r}_{5})}({\bf{X}},t) & neurons=1000 & \begin{array}{cc}{\rm{P}}{\rm{e}}{\rm{r}}{\rm{i}}{\rm{o}}{\rm{d}}\,5: & 100 < t\le 200\end{array}\\ {C}^{({r}_{6})}({\bf{X}},t) & neurons=1000 & \begin{array}{cc}{\rm{P}}{\rm{e}}{\rm{r}}{\rm{i}}{\rm{o}}{\rm{d}}\,6: & 200 < t\end{array}\end{array},$$

where **X** is the acoustic features matrix over time and *t* is the epoch’s index.

Each *sub-classifier* $({C}^{({r}_{2})}\ldots {C}^{({r}_{6})})$ is configured with input neurons, *tangent sigmoid* hidden layer, and *softmax* output layers.

Period 1: Epochs 1 to 5 are considered to be a “Wake” state regardless of the features values. This arbitrary decision (“Wake”) is reasonable since in most of the cases, audio recording begins with lights off when the subject is still awake.

Period 2: Epochs 6 to 20, estimated based on the five previous epochs’ features values.

Period 3: Epochs 21 to 50, estimated based on the 20 previous epochs’ features values.

Period 4: Epochs 51 to 100, estimated based on the 50 previous epochs’ features values.

Period 5: Epochs 101 to 200, estimated based on the 100 previous epochs’ features values.

Period 6: Epochs 201 to ∞, estimated based on the 200 previous epochs’ features values.

The sub-classifiers epochs’ intervals were defined arbitrarily to capture short to long temporal relations, and up to REM cycles (usually 90–100 minutes, i.e., 180–200 epochs). Supplementary Fig. S9 shows a diagram of the classifier configuration. We constructed this classifier setup so we can utilize enough information prior to the estimated epoch, yet making the classifier simple for computation and convergence.

#### Offline MSS classification

There are cases where a general sleep evaluation report is required, meaning real-time estimation is not necessary. Off-line MSS evaluation might produce higher MSS accuracy by applying additional knowledge of standard sleeping pattern and the knowledge of “future” MSS estimations. The additional sleep pattern knowledge was inserted using an additional (offline) classifier (*C*^(*o*)^), which runs backward in time on the real-time MSS estimations. The offline classifier was composed of six sub-classifiers adequate for six different periods (period 1–6):

$${C}^{(o)}({C}^{(r)},t)=\{\begin{array}{ccc}{C}^{({o}_{1})}({C}^{(r)}({\bf{X}},t),t) & neurons=0 & \begin{array}{cc}{\rm{P}}{\rm{e}}{\rm{r}}{\rm{i}}{\rm{o}}{\rm{d}}\,1: & T-5\le t\end{array}\\ {C}^{({o}_{2})}({C}^{(r)}({\bf{X}},t),t) & neurons=20 & \begin{array}{cc}{\rm{P}}{\rm{e}}{\rm{r}}{\rm{i}}{\rm{o}}{\rm{d}}\,2: & T-20\le t < T-5\end{array}\\ {C}^{({o}_{3})}({C}^{(r)}({\bf{X}},t),t) & neurons=50 & \begin{array}{cc}{\rm{P}}{\rm{e}}{\rm{r}}{\rm{i}}{\rm{o}}{\rm{d}}\,3: & T-50\le t < T-20\end{array}\\ {C}^{({o}_{4})}({C}^{(r)}({\bf{X}},t),t) & neurons=100 & \begin{array}{cc}{\rm{P}}{\rm{e}}{\rm{r}}{\rm{i}}{\rm{o}}{\rm{d}}\,4: & T-100\le t < T-50\end{array}\\ {C}^{({o}_{5})}({C}^{(r)}({\bf{X}},t),t) & neurons=200 & \begin{array}{cc}{\rm{P}}{\rm{e}}{\rm{r}}{\rm{i}}{\rm{o}}{\rm{d}}\,5: & T-200\le t < T-100\end{array}\\ {C}^{({o}_{6})}({C}^{(r)}({\bf{X}},t),t) & neurons=200 & \begin{array}{cc}{\rm{P}}{\rm{e}}{\rm{r}}{\rm{i}}{\rm{o}}{\rm{d}}\,6: & 0 < t < T-200\end{array}\end{array},$$

where *T* is the total number of epochs.

Period 1: The five last epochs are considered to be the estimation of the realtime classifier, and used to be the starting point for the next sub-classifier.

Period 2: Epochs 6 to 20 from the end, estimated based on the five future epochs’ features values.

Period 3: Epochs 21 to 50 from the end, estimated based on the 20 future epochs’ features values.

Period 4: Epochs 51 to 100 from the end, estimated based on the 50 future epochs’ features values.

Period 5: Epochs 101 to 200 from the end, estimated based on the 100 future epochs’ features values.

Period 6: Epochs 1 up to 201 from the end, estimated based on the 200 future epochs’ features values.

### Sleep evaluation report

Using the estimated MSS pattern, eight sleep quality parameters were calculated, including 1) Total sleep time (TST) – actual sleep time in bed, i.e., recording time, equal to the amount of REM-sleep and NREM-sleep combined. 2) Sleep latency (SL) – period measured from “lights out”, or bedtime, to the beginning of sleep. We measured from the start of the recording, assuming recording was started when the subject was in bed. 3) Sleep efficiency (SE) – the ratio of total sleep time to the overall time in bed. 4) Wake time after sleep onset (WASO) – the overall time spent awake after sleep has been initiated and before final awakening. 5) REM latency (RL) – period measured from “lights out”, or bedtime, to the beginning of REM-sleep. 6) REM percentage (RP) – The ratio of total REM periods to the total sleeping time. 7) NREM percentage (NP) – The ratio of total NREM periods to the total sleeping time. 8) REM cycles (RC) – The number of REM cycles presented in the TST. For more detail regarding REM cycles calculation, see “Sleep evaluation report” section in Supplementary information.

### Data and statistical analyses

Audio signal processing and statistical analyses were performed using MATLAB (R-2016b, The Mathworks, Inc., Natick, MA, USA). A sample size of 92 subjects was calculated to provide a statistical power of 0.80 (*α* = 0.05) to achieve > 0.7 Cohen’s kappa agreement for three states (MSS) estimation for each subject. Therefore, 100 subjects were recruited for the validation study. PSG, demographic, and audio data were compared between design and validation study groups using unpaired two-tailed Student *t*-test or χ^2^ test. Two confusion matrices were calculated on the validation dataset to produce epoch-by-epoch MSS comparison, one for real-time and one for offline. To assess how parameters such as age, gender, BMI, AHI, ESS, SE, and respiration SNR affected the performance of our proposed system, we tested the Pearson correlation coefficient for each of these parameters and ran a multivariate analysis using linear regression.

In the analyses, first, for each subject in the validation dataset, we performed epoch estimation performances as described above. A global average, standard deviation, and 95% confidence interval were calculated among all validation subjects. Second, based on the estimated epochs, for each subject, we calculated the eight sleep quality parameters and compared to PSG. Concordance correlation, average error (absolute differences), and relative error were calculated between the two approaches. Figure 2 presents a visualization of subject’s sleep quality parameters. For more information regarding SSA overfitting to the patient’s data, please see Supplementary “Overfitting assessment” section.

## Electronic supplementary material

Supplementary Information

## Data Availability

Patient data are not publicly available due to ethics restrictions.
