# Supplementary material for: Sleep staging using nocturnal sound analysis
Source: Sci Rep. 2018 Sep 7;8:13474. doi: 10.1038/s41598-018-31748-0 (PMC6128888; doi:10.1038/s41598-018-31748-0)
Supplement: Supplementary file 1 — Supplementary Information [file 41598_2018_31748_MOESM1_ESM.pdf]

Supplementary Information

Sleep staging based on nocturnal sound analysis

Eliran Dafna[1,*], Ariel Tarasiuk[2], Yaniv Zigel[1]

[1] Department of Biomedical Engineering, Faculty of Engineering, Ben-Gurion University of the Negev, Beer–Sheva, Israel; e-mail: elirandafna@gmail.com, yaniv@bgu.ac.il

[2] Sleep-Wake Disorders Unit, Soroka University Medical Center, and Department of Physiology, Faculty of Health Sciences, Ben-Gurion University of the Negev, Israel; e-mail: tarasiuk@bgu.ac.il

[*] Corresponding author: Eliran Dafna, e-mail: elirandafna@gmail.com

Results

Subjects characterization and sleep patterns

Supplementary Table S1. Subject anthropometric parameters.

	System Design (*n*=150)	System Validation (*n*=100)	*p*
Gender (M/F)	97/53	65/35	.957
Age (yr)	52.5±15.5<br>(23–81)	52.9±16.9<br>(19–84)	.862
BMI (kg/m^2)	30.7±5.6<br>(21.6–45.1)	31.4±6.3<br>(21.8–46.8)	.306
ESS (score)	9.6±5.6<br>(0–23)	10.1±5.6<br>(1–21)	.495
Total AHI (events/hr)	16.0±14.3<br>(1.4–58.8)	19.2±16.5<br>(1.3–56.8)	.108
REM AHI (events/hr)	21.8±21.7<br>(0.0–76.6)	24.9±24.9<br>(0.0–96.5)	.298
NREM AHI (events/hr)	15.2±14.4*<br>(0.0–54.4)	17.7±15.4*<br>(0.0–57.1)	.192
Wake percentage (%)	18.6±11.2<br>(3.7–47.3)	19.2±13.6<br>(3.8–52.2)	.704
REM percentage (%)	11.1±6.7<br>(0.0–26.9)	10.7±7.8<br>(0.0–27.8)	.666
NREM percentage (%)	70.3±10.6<br>(48.2–89.6)	70.1±11.5<br>(43.5–87.5)	.888

M – male; F – female; BMI – body mass index; ESS – Epworth sleepiness scale; REM – rapid eye movement; NREM – non-rapid eye movement; AHI – apnea-hypopnea index. Values are mean ± SD (95% CI); *p*-value was calculated using an unpaired t-test for age, BMI, ESS, and AHI; and χ^2 for gender. Wake, REM, and NREM percentages are calculated from the entire sleep recording of a subject.
* <0.005 comparing NREM to REM AHI by paired t-test.

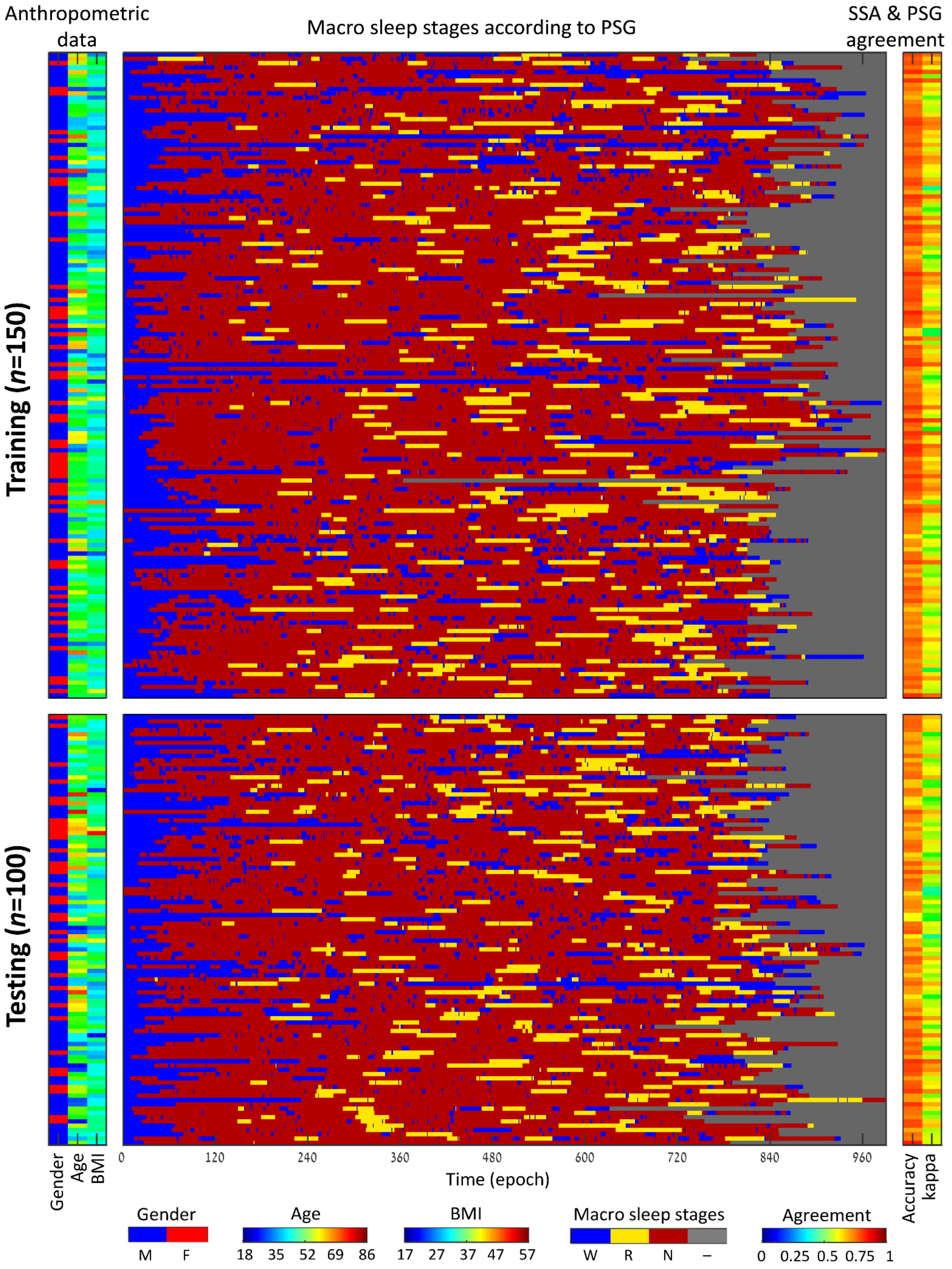

Supplementary Fig. S1 | Individual big-data visualization for the study design and validation. A – Study design dataset (training, *n*=150); **B** – Validation dataset (testing, *n*=100). Each horizontal line represents individual data. Sleep stages were manually scored epoch-by-epoch (30 sec) using the polysomnography (PSG) data. Note the large individual differences in sleep stages. The onset of the gray area indicates study termination for each subject. Accuracy and Cohen's kappa coefficient epoch-by-epoch of sleep stages for each subject were calculated comparing the proposed sleep sound analysis (SSA) system and the gold standard PSG; BMI – body mass index. For study protocol, see main body of the manuscript.

Macro sleep stages estimation

Fig. S2 shows the detection (precision and recall) of each MSS (wake, REM, and NREM) among subjects for both realtime and offline estimation.

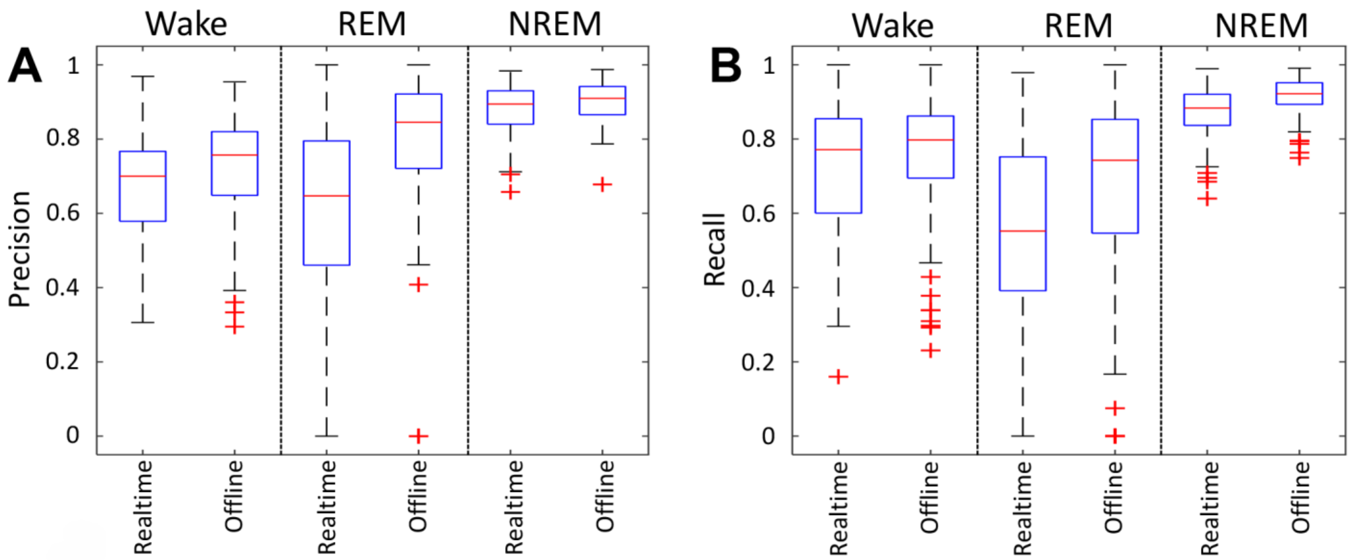

Supplementary Fig. S2 | Macro sleep stages detection. Presented here boxplots of macro sleep stages (wake, REM, and NREM) detection in manner of precision (**A**) and recall (**B**) among subjects in the validation dataset.

Offline system performances for each subject can be seen in **Fig. S1** rightmost columns. Additionally, performance of given epoch estimation (wake, REM, and NREM) was measured as a function of the subject-induced sounds such as respiratory and body movement sounds, relative to the background noise level (signal to noise ratio, SNR) of the testing room in the sleep laboratory using the validation dataset. In our setting, the average SNR overnight among subjects ranged from -18.3 dB to 2.7 dB (95% CI). We found that the estimation accuracy of a given epoch improved by 2.2% for every 10 dB increase in SNR.

System accuracy of offline MSS estimation was analyzed across subjects' anthropometric characteristics (**Supplementary Fig. S3A–C**), AHI, and sleep efficiency (**Supplementary Fig. S3D,E**) using the validation dataset. Univariate analysis revealed that accuracy inversely correlates with age, BMI, and AHI; and sleep efficiency positively correlates with system accuracy. Multivariate analysis revealed that only sleep efficiently correlates with system accuracy (adjusting for gender, age, BMI, AHI, and sleep efficiency) (**Supplementary Fig. S3F**). For every 10% increase in sleep efficiency, system MSS accuracy increases by 1.3%.

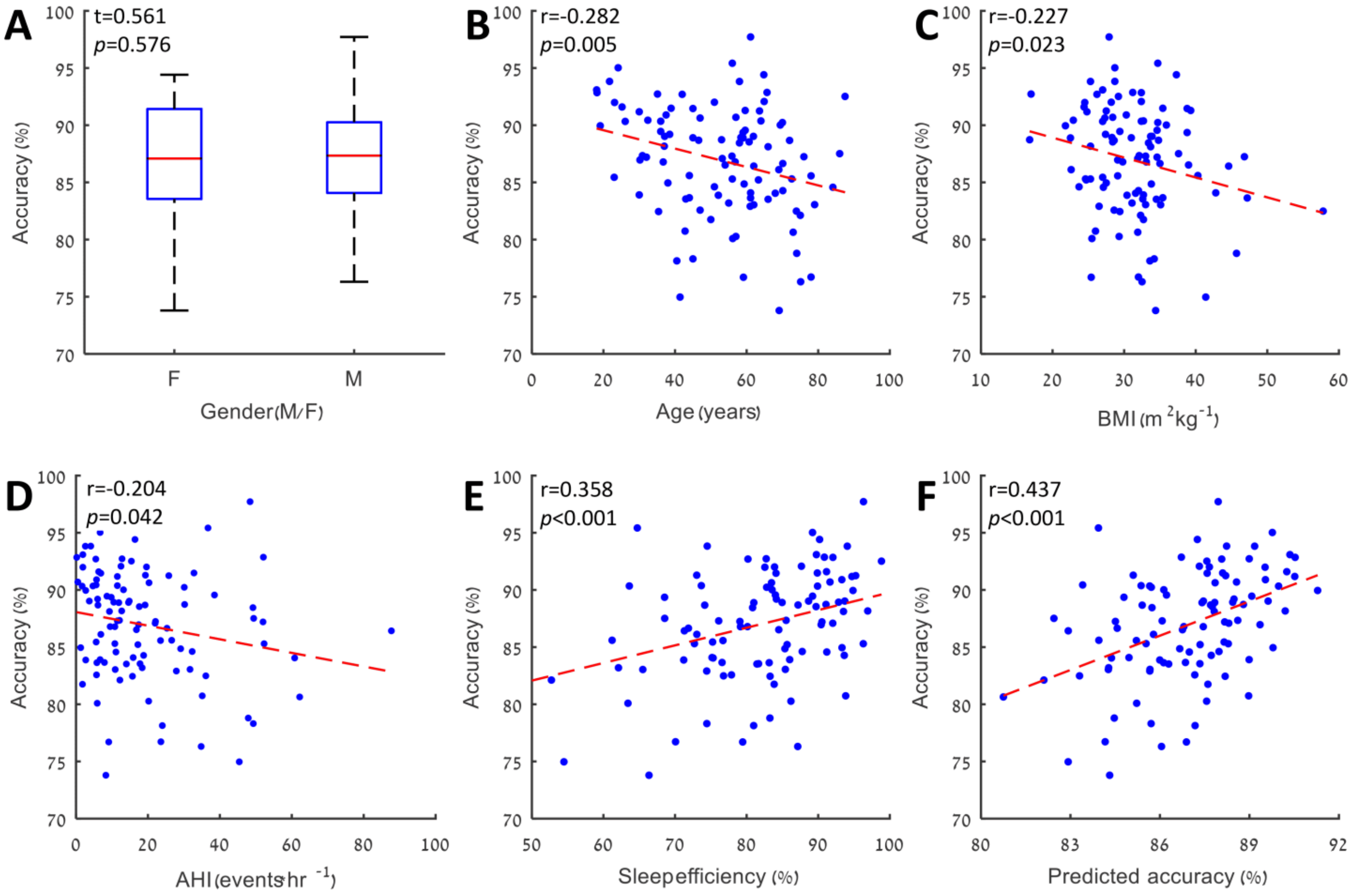

Supplementary Fig. S3 | Association between system performance and subject characteristics. System accuracy was calculated epoch-by-epoch between polysomnography and sleep sound analysis (SSA) using validation dataset (*n*=100). **A**) Gender, showing boxplot, measuring the quartile distribution of accuracy agreements between genders; B–E showing the Pearson correlation between SSA and Age (**B**); Body mass index (BMI) (**C**); Apnea-hypopnea index (AHI) (**D**), and Sleep efficiency (SE) (**E**). **F**) A multivariate regression analysis between predicted SSA based on subject characteristics (gender, age, BMI, AHI, and SE) and system accuracy. Each dot represents one individual from the validation dataset (*n* = 100); r – is the regression coefficient and its *p*-value.

Sleep quality parameters

Using the detected MSS from the offline analysis, sleep quality parameters were calculated. Comparison between SSA estimation and PSG is presented using Bland-Altman plot. Future studies are needed to validate our approach on narcolepsy or insomnia patients.

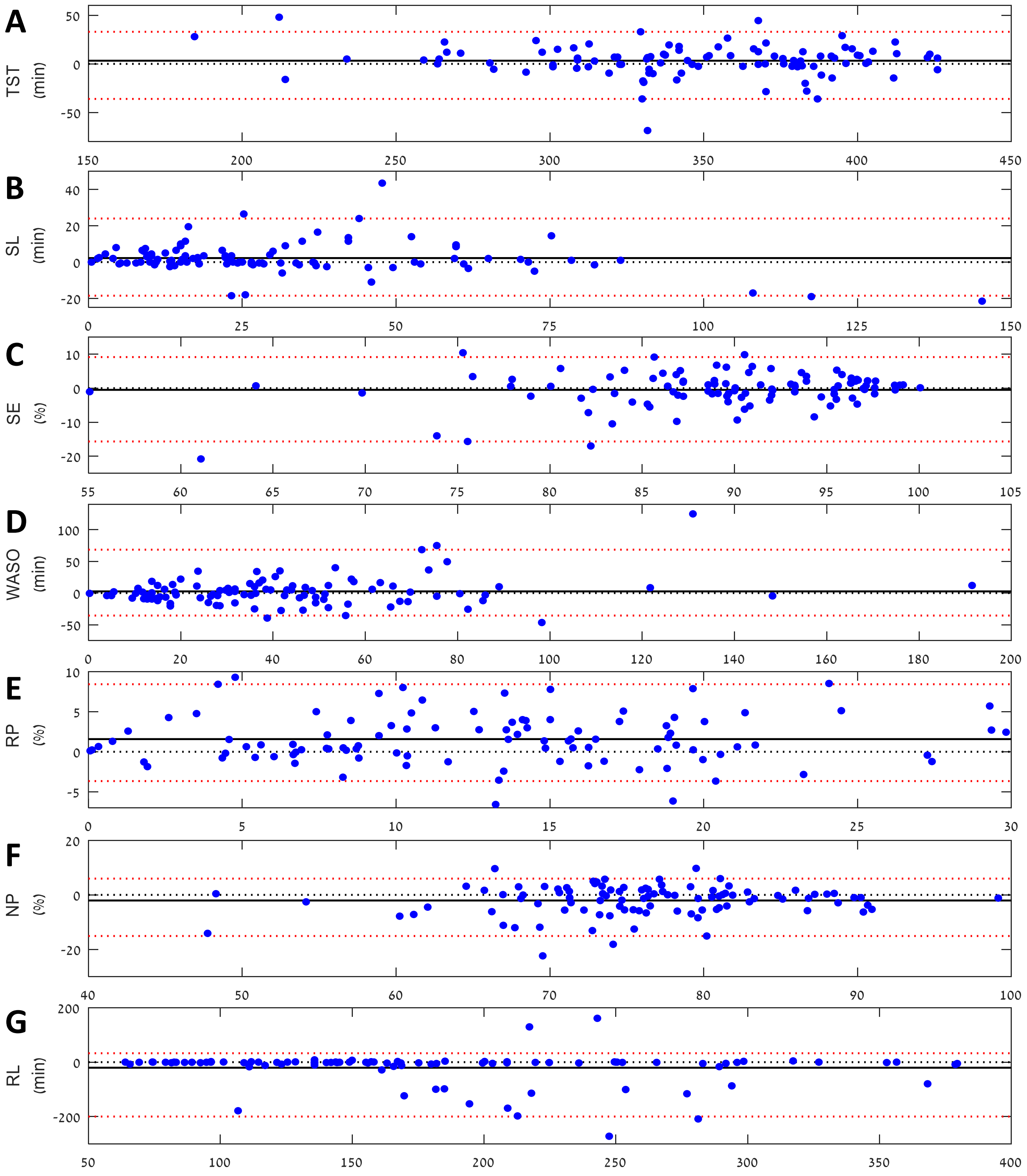

Supplementary Fig. S4. Bland-Altman plot of sleep parameters. A) TST – Total sleep time; **B**) SL – Sleep latency; **C**) SE – Sleep efficiency; **D**) – Wake after sleep onset; **E**) RP – Rapid-eye-movement percentage of total sleep time; **F**) NP – Non-rapid-eye-movement percentage of total sleep time; **G**) RL – REM latency; Data was taken from the validation dataset (*n*=100), each data point represents a subject; Black dashed line – mean difference between polysomnography (PSG) and sleeping sound analysis (SSA); X-axis represents the mean sleep parameter value between the polysomnography (PSG) and breathing sound analysis (BSA) in the relevant parameter units. The Y-axis is the difference between the PSG and BSA sleep quality parameter (SSA-PSG). The dashed lines represent the 95% CI for the scatter.

The sound of sleep

Sleeping sounds include several types of sounds from several sources including vocal sounds, body frictions (body movements), and other sounds such as clock ticking, barking dogs. In this study, we grouped those into three main sources: 1) breathing sounds; 2) body movement sounds, and 3) other sounds. **Fig. S5** shows the distribution of sleeping sound sources and their sound intensities (volume) during the night. Manual annotations and segmentations were conducted by several raters using ad hoc graphical user interface (GUI), which involved hearing and visualization of several PSG channels (including effort belts, and EMG) along with spectrogram of the audio (for each epoch). Annotations were made at 50 ms resolution.

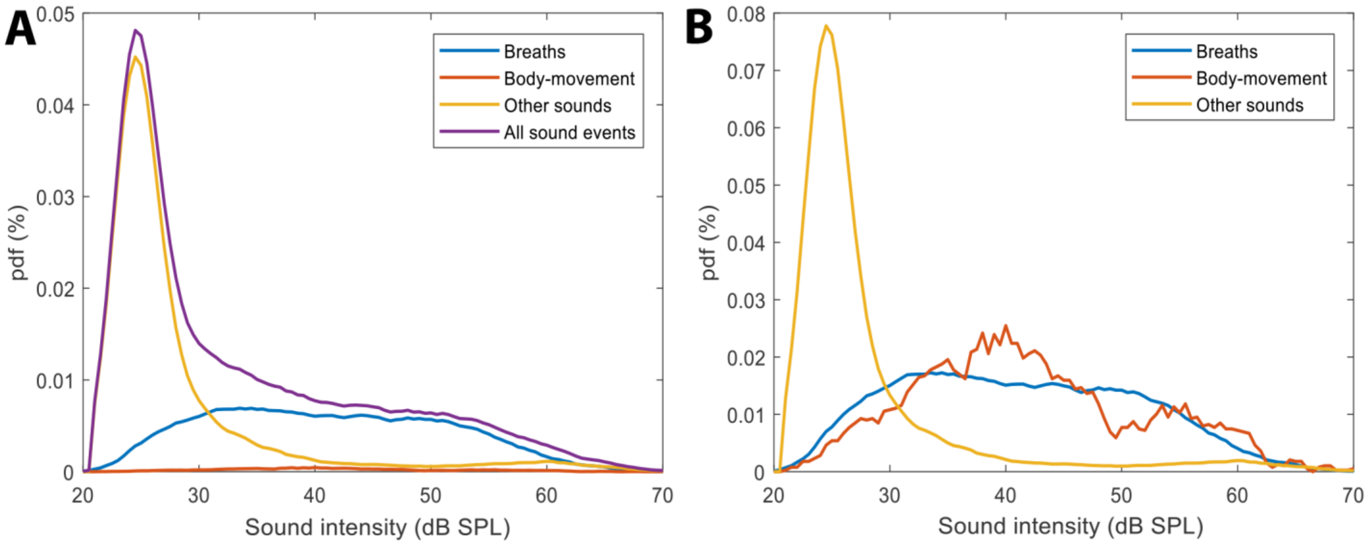

Supplementary Fig. S5 | Sleeping sounds distribution. A) Sleeping sound content and sound intensity in dB SPL (sum of all sources across all dBs gives 100%); **B**) Distribution of each sound source according to the sound intensity (sum of each sound source across all dBs gives 100%). Data were collected from 67 patients that underwent full manual annotation of sleeping sounds (into three classes: breaths, body movements, and other sounds). Pdf – probability density function.

Detectors robustness estimation

Sleeping sounds include several sources including vocal sounds, body movements (frictions), and other. **Fig. S6** shows the performances of the detectors as a function of the signal quality (SNR). To measure the performance of specific SNR value, sound events from all subjects were sorted and divided into 4 dB sub-bands with 50% overlaps.

Detection agreement of a sound event (segmentation and detection) was measured by comparing frame-by-frame (50 ms resolution) manual annotation with the detector's predictions. Although this comparison is extremely strict, e.g., detection of 1.0-second event (20 frames) at 50 ms delay (1 frame) will results in 19/21 (90%) frames agreement, in this study, we chose this comparison method because it measures the quality of both segmentation and detection, and it is easy to implement as classifier cost function. Detectors were designed based on 25 subjects and were validated on a 42 subjects dataset.

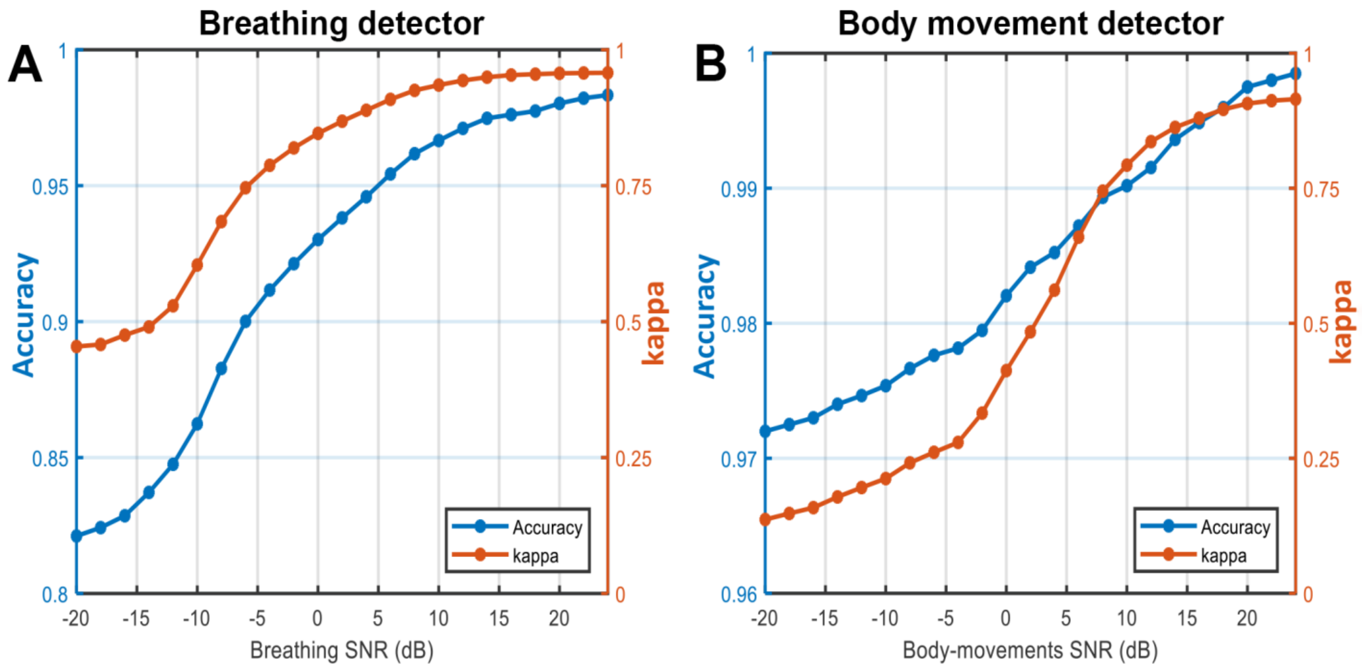

Supplementary Fig. S6 | Breathing and body movement detectors – Performance vs. SNR. A) Breathing detector (inhalation/ exhalation/ non-breathing) accuracy and Cohen's kappa coefficient; **B**) Body movement detector (body-movement/ non-body-movement) accuracy and Cohen's kappa coefficient. In each plot, the right Y-axis represents the accuracy scale (blue curve), and the left Y-axis represents the Cohen's kappa coefficient value (red curve); please note the different Y-axis scales. Data were collected from 42 patients that underwent full night manual annotation of sleeping sounds.

Assessing feature importance

To assess the importance of each feature in the classifier, we measured the impact of the MSS classification (Cohen's kappa coefficient) when corrupting only the tested feature.

The corruption was achieved by scrambling (permutation) the feature values along the time index (for each sleep sequence). In order to maintain valid values for the feature (and the classifier), we repeated the corruption for each period suitable for each sub-classifier. To minimize the randomness effect, we averaged the scores of 30 permutations for each test feature/s.

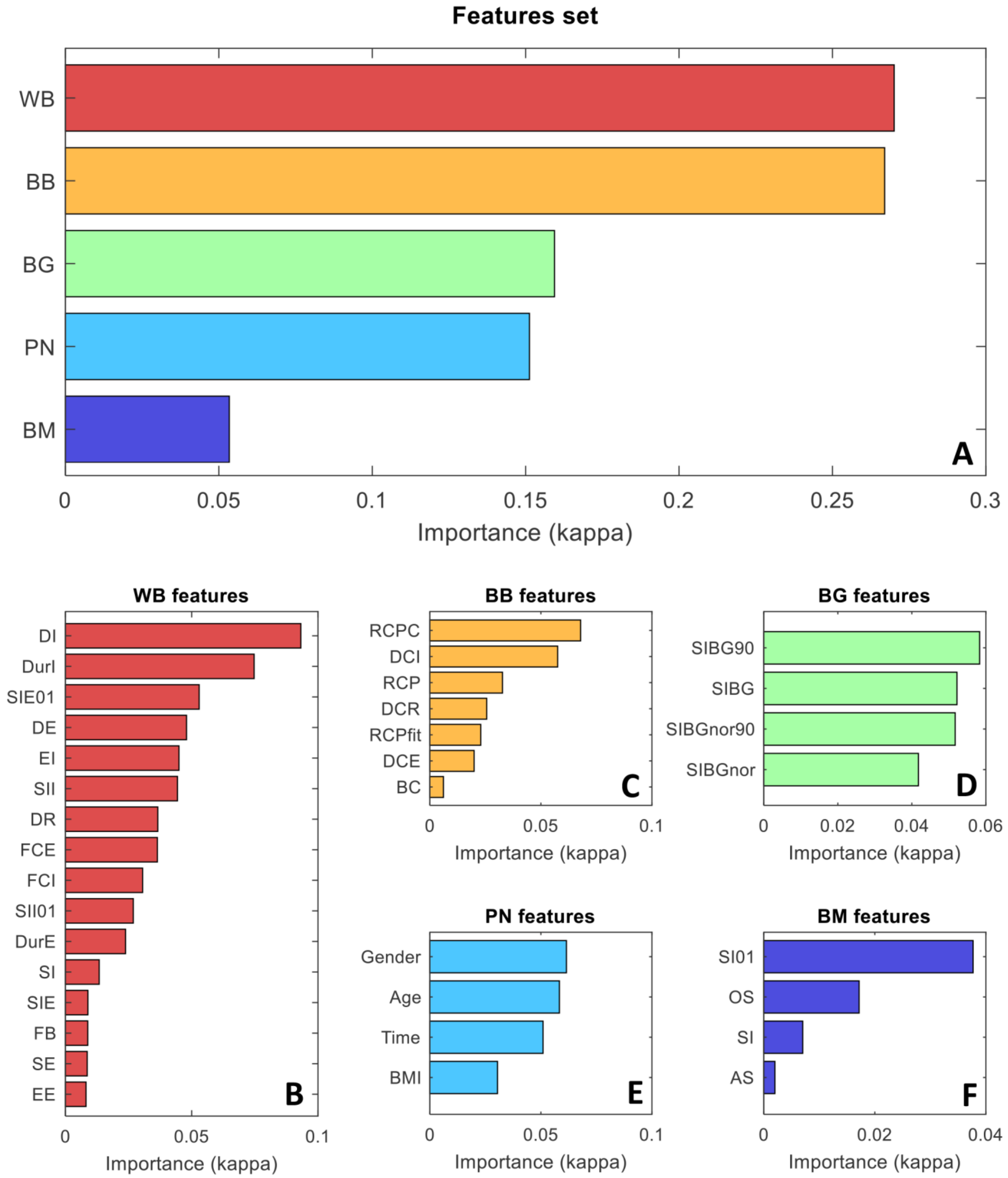

Supplementary Fig. S7. | Feature importance. Feature importance was estimated by measuring the impact on performance when feeding a permutated version of the tested feature (permutation along time step) into the MSS classifier. The importance values are presented as a horizontal bar chart to visualize the relative improvement between features. **A)** Features set – each feature category set; **B)** WB – within-breathing features; **C)** BB – between-breathing features; **D)** BG – background noise features; **E)** PN – personalization features; and **F)** BM – body-movement features. The importance value represents the degradation in MSS classification (kappa coefficient) from the complete model (reference kappa = 0.694), e.g., without the within-breathing-features set (WB) system performance was degraded to 0.424 (-0.270).

Methods

Sleep sound analysis system

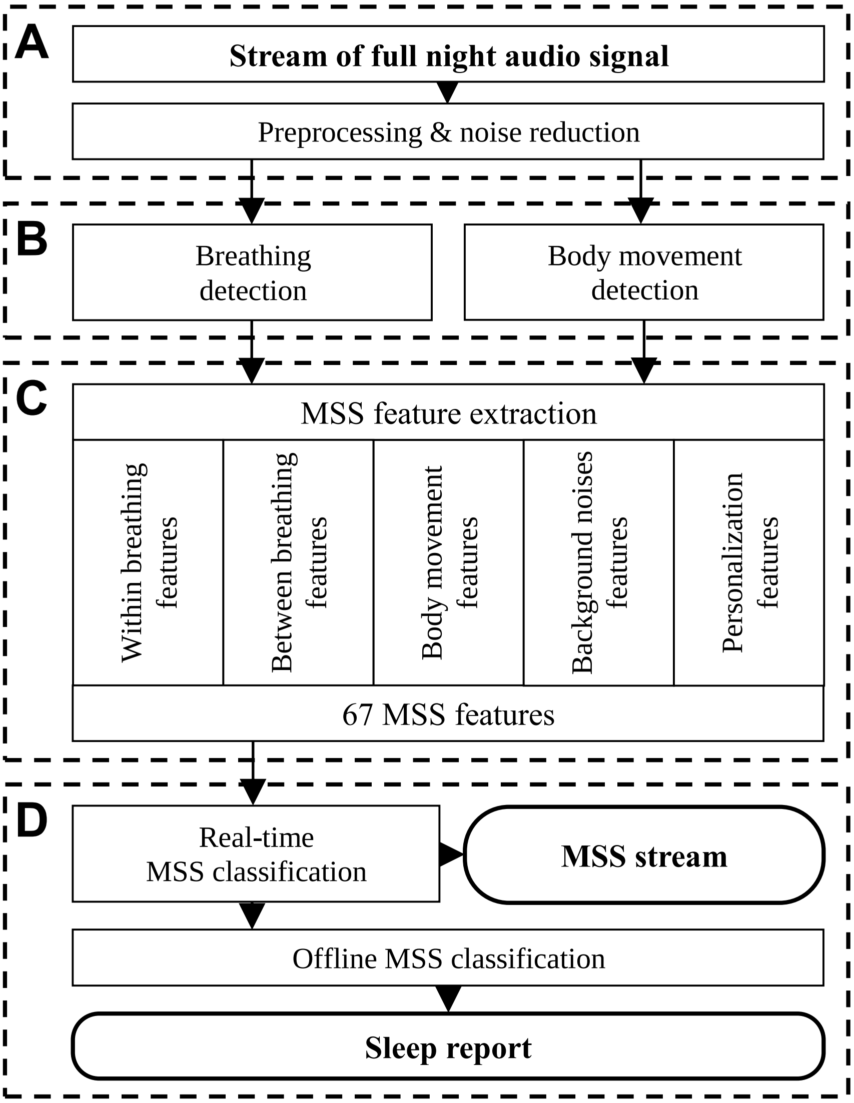

Supplementary Fig. S8 | Block diagram of the proposed system. The system is composed of four main stages: A) pre-processing and noise reduction, B) breathing and body movement detection, C) Feature extraction, D) MSS classification. Whole night audio signals were recorded using a non-contact microphone. To simulate real-time analysis (at 30-sec resolution), data was fed using streaming protocol. The outputs of the system are MSS stream – a real-time (stream) estimation at 30-sec epoch resolution, and Sleep report – Sleep quality parameters calculated using the offline 30-sec MSS estimation.

Feature extraction

Supplementary Table S2. Features pool.

Feature	Symbol	Count	Importance
**A. Within breathing features (WB)**	**Feature code**	**33**	**0.270**
Detection score of inspiration (μ,σ)	WB_DI	2	0.093
Detection score of expiration (μ,σ)	WB_DE	2	0.048
Detection score of respiration (μ,σ)	WB_DR	2	0.037
Duration inspiration (μ,σ)	WB_DurI	2	0.075
Duration expiration (μ,σ)	WB_DurE	2	0.024
Stationarity inspiration (μ,σ)	WB_SI	2	0.013
Stationarity expiration (μ,σ)	WB_SE	2	0.009
Sound intensity inspiration (μ,σ)	WB_SII	2	0.044
Sound intensity expiration (μ,σ)	WB_SIE	2	0.009
Sound intensity inspiration top 1% (μ,σ)	WB_SII01	2	0.027
Sound intensity expiration top 1% (μ,σ)	WB_SIE01	2	0.053
Entropy inspiration (μ,σ)	WB_EI	2	0.045
Entropy expiration (μ,σ)	WB_EE	2	0.008
Frequency centroid inspiration (μ,σ)	WB_FCI	2	0.031
Frequency centroid expiration (μ,σ)	WB_FCE	2	0.036
Frequency bandwidth (resp., insp., expi.)	WB_FB	3	0.009
**B. Between breathing features (BB)**		**12**	**0.267**
Respiration duty cycle	BB_DCR	1	0.026
Inspiration duty cycle	BB_DCI	1	0.058
Expiration duty cycle	BB_DCE	1	0.020
Respiration cycle period (μ,σ)	BB_RCP	2	0.033
Respiration cycle period consistency	BB_RCPC	1	0.068
Respiration cycle periods fourth-order curve	BB_RCPfit	5	0.023
Breathing Count	BB_BC	1	0.006
**C. Body movement features (BM)**		**10**	**0.054**
Body movement average score	BM_AS	1	0.002
Body movement overall score percentiles	BM_OS	7	0.017
Sound intensity body movement (all curve)	BM_SI	1	0.007
Sound intensity body movement 10% (all curve)	BM_SI01	1	0.038
**D. Background noises features (BG)**		**8**	**0.159**
Sound intensity background (μ,σ)	BG_SIBG	2	0.052
Sound intensity background 90% (μ,σ)	BG_SIBG90	2	0.058
Sound intensity background normalized(μ,σ)	BG_SIBGnor	2	0.042
Sound intensity background 90% normalized (μ,σ)	BG_SIBGnor90	2	0.052
**E. Personalization features (PN)**		**4**	**0.151**
Subject's age (years)	PN_Age	1	0.058
Subject's gender (1-M, 2-F)	PN_Gender	1	0.062
Subject's BMI ($kg*m^{-2}$)	PN_BMI	1	0.031
Epoch's time index (log scale)	PN_Time	1	0.051

Table presents the name of the feature, its symbol, number of features used (count), and its importance.
The code name (symbol) for each feature is composed of prefix (symbol group family) and suffix (individual symbol abbreviation). For example, WB_SIE01, i.e., meaning within breathing feature (WB) indicating sound intensity expiration top 1%. μ – mean; σ – standard deviation. The importance value represents the decrement in MSS classification (kappa coefficient) from the complete model (reference kappa = 0.694), e.g., without the within-breathing-features set (WB) system performance is degraded to 0.424 (-0.270).

Feature calculations

The following section describes how to calculate some of the features presented in Table S2.
Denote $\mathbf{S}_{ft}$ as the spectrogram of the epoch being tested (half-sided FFT 512 coefficients, 50 ms resolution, 50 ms frame rate, 30 s epoch, 600 frames per epoch), for convenience the values are stored in logarithmic scale.

Denote F_t as the frames $F_t = \sum_{f=0}^{Fs/2} \mathbf{S}_{ft}$.

Denote $D_t^{In}, D_t^{Ex}, D_t^{BM}, D_t^{R} = D_t^{In} + D_t^{Ex}$ as the detectors curves (Figure 4D main body) for detecting inhale, exhale, body movements, and respiration; these values are detection probabilities (likelihood scores), and between 0 and 1, higher scores represent "detection".

- WB_DI**,** WB_DE, WB_DR are calculated as the mean (and std) of the detected curves (*D*) in the epochs.
- WB_DurI**,** WB_DurE – duration in seconds for the detected breathing events. Calculation yields mean and std of the breathing duration.
- WB_SI**,** WB_SE – the mean (and std) of breaths in a manner of stationarity. The stationarity measurement was calculated as the differences between frames (50 ms) of the breathing normalized by the total epoch's energy.
- WB_SII**,** WB_SIE, WB_SII01, WB_SIE01 were calculated based on the estimated sound intensity (in dB) of each frame measured from $\mathbf{S}_{ft}$.
- WB_EI, WB_EE were calculated as the entropy of each frame from $\mathbf{S}_{ft}$.
- WB_FCI, WB_FCE were calculated as: $WB\_FC_t = \left(\sum_{f=0}^{Fs/2} f \times \mathbf{S}_{ft} \right) \times F_t^{-1}$
- WB_FB was calculated as: $WB\_FB_t = \frac{1}{256} \sum_{f=0}^{Fs/2} \mathrm{Bool}\left(\mathbf{S}_{ft} > F_t / 2 \right)$
- BB_DCR, BB_DCI, BB_DCE were calculated as: $BB\_DC = \frac{1}{600} \sum_{t=1}^{600} \mathrm{Bool}\left(D_t > 0.5 \right)$.
- BB_RCP, BB_RCPC were calculated by estimation of breathing period using autocorrelation function.
- BB_RCPfit – fourth order curve fit for breathing period along the epoch.
- BM_AS was calculated as: $BM\_AS = \frac{1}{600} \sum_{t=1}^{600} \mathrm{Bool}\left(D_t^{BM} > 0.5 \right)$
- BM_OS was calculated as the D_t^{BM} percentile at 80, 85, 90, 95, 97, 98, and 99%.
- BM_SI was calculated as: $BM\_SI = \left(\sum_{t=1}^{600} \mathrm{Intensity}\left(D_t^{BM} > 0.5 \right) \right) \times \left(\sum_{t=1}^{600} \mathrm{Bool}\left(D_t^{BM} > 0.5 \right) \right)^{-1}$
- BM_SI was calculated as: $BM\_SI01 = \mathrm{Intensity}\left(D_t^{BM} > 0.5 \right)\Big|_{10\%\ percentile}$
- BG_SIBG was calculated as the mean (and std) of the sound intensity where background noise is detected.
- BG_SIBG90 same as BG_SIBG but calculated over the 90-100% percentiles.
- BG_SIBGnor – same as BG_SIBG but was normalized by the frame with the maximum sound intensity.
- BG_SIBGnor90 – same as SIBG90 but was normalized by the frame with the maximum sound intensity.

MSS Classifier

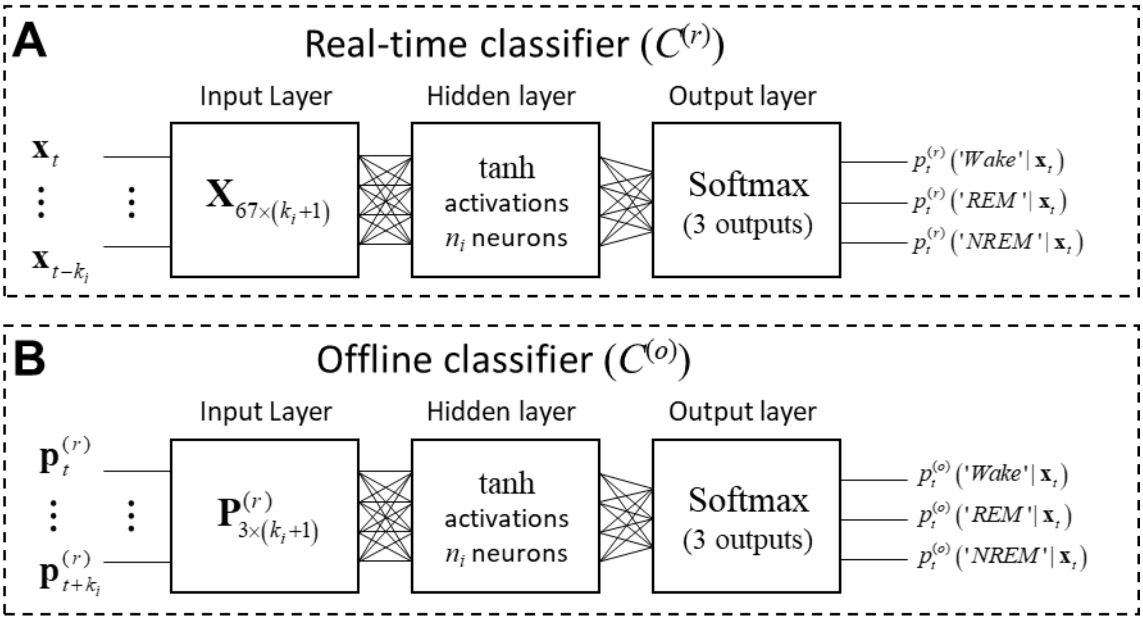

Supplementary Fig. S9 | Classifier configuration. A) Real-time classifier ($C^{(r)}$); **B**) Offline classifier ($C^{(o)}$). The input for the $C^{(r)}$ is k_i time step series of acoustic features (**x**). The output of $C^{(r)}$ is the estimation of the current epoch (t) using three MSS probability scores ($\mathbf{p}^{(r)}_t$). The input of the offline classifier $C^{(o)}$ is the real-time classifier output ($\mathbf{p}^{(r)}_t$) at different time-steps (future epochs) to produce three MSS probability scores ($\mathbf{p}^{(o)}_t$).

Training the MSS classifiers

In this work, we configured time-series classifiers that are designed to learn short- to long-term relations between epochs, i.e., from adjacent epochs' relations and up to the relations between two REM cycles of roughly 90-100 minutes (180-200 epochs). We chose to work with one hidden-layer ANN as it was powerful enough to learn the discriminative information on the design dataset features, yet simple enough to overcome the "overfitting curse" on a small database. In our case, 100 subjects (out of 150 subjects) on the design dataset had a simultaneous recording from two audio recorders, therefore presenting 200 observations for each time-step. We treated the additional records as new subjects, i.e., a total of 250 records in the design dataset; although it is not good as 250 different subjects, it is better than 150 in the sense of model convergence and robustness to different microphones specifications, distance and angle to subject's head. Another challenging task in the design phase was the unbalanced MSS class sizes, e.g., NREM is more abundant than REM and wake during sleep. To overcome this, we formulated a penalty weight for each epoch proportional to the PSG a priori probability of each MSS, as follows in Eq. 1

$$Weight_i = \frac{N}{\sum_{j=1}^{N} \mathrm{Bool}\left(Epoch_j = Epoch_i\right)}, \quad Epoch_i \in \{Wake, REM, NREM\} \tag{1}$$

where N is the total number of epochs for the design dataset, and *Bool* is the Boolean operator resulting in "1" if the statement is true. In recent studies[1-3] it has been shown that recurrent neural network (RNN) and long-short-term memory (LSTM) neural network have superior potential to learn the relations found in time-series data.

In our ongoing attempts to a bi-directional LSTM (BiLSTM) model as a classifier, we achieved almost similar, yet slightly inferior, results for three-class estimations, mainly due to REM misdetection (see **Fig. S10**). We hypothesized that similar to BiLSTM, our original proposed classifier holds enough information

presented in both the short- and long-term memory for past and future information (using up to 200 adjacent epochs each side). Consequently, training each sub-classifier (separately) proved to be a much easier task (to converge) with less sensitivity to hyperparameters values compared to the BiLSTM model. Further studies are needed to support these findings. Presented here is our attempt to use the BiLSTM model.

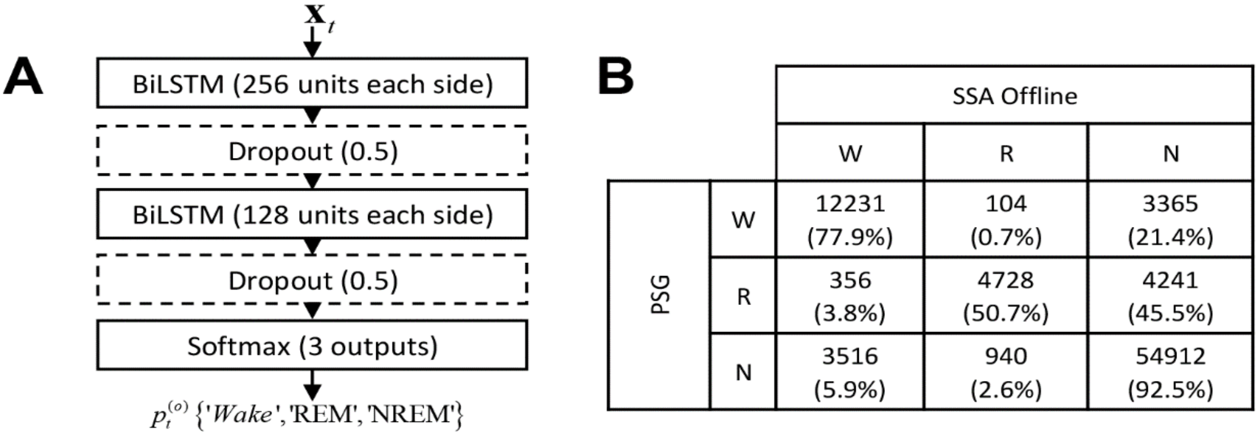

Supplementary Fig. S10 | BiLSTM classifier configuration and performance. A) Model configuration; **B**) confusion matrix for offline MSS classification. Performances are 85.1% accuracy and a kappa coefficient of 0.66.

Overfitting assessment

Several aspects are affecting system performances including microphone specifications, distance to subject's head, bedroom background noise level, and even subject's loudness. In our study, most aspects were fixed except the sounds (intensities) generated by the subjects. To overcome some of these aspects, we trained our model using a large database and two types of microphones (see main body for more information) located at a 90 degrees angle and about 0.5 – 1.0 m distance to the subject's head.

Robustness of the model can be indicated by achieving for each subject high agreement with the gold standard (PSG), and with a minor deviation between subjects. Additionally, performances of the training dataset may imply the upper-limit performances while large differences between training and validation datasets may imply an "overfitting" situation. **Table S3** summarizes performances of the training and validation datasets.

Supplementary Table S3. System design and validation performances.

		**Accuracy (%)**		**Cohen's kappa (κ)**	
**Protocol**	**Dataset**	**Mean ± SD**	**Median (95% CI)**	**Mean ± SD**	**Median (95% CI)**
Real-time	Design	85.5±5.8	86.7 (70.7–94.2)	.654±.126	.674 (.351–.854)
	Validation	82.2±6.4	82.6 (69.0–93.3)	.590±.122	.598 (.323–.798)
	Difference	3.3 (*p*<0.0001)		.064 (*p*<0.0001)	
Offline	Design	89.7±4.2	90.3 (79.3–96.0)	.748±.114	.766 (.444–.911)
	Validation	86.9±4.8	87.3 (76.3–95.0)	.694±.113	.700 (.377–.869)
	Difference	2.8 (*p*<0.0001)		.054 (*p*<0.0001)	

p-value is calculated for unpaired t-test two-tailed (250 and 100 samples for design and validation, respectively).

One can see minor differences between design and validation datasets (~3% and κ=.06).

Sleep evaluation report

REM cycles (RC) and REM latency (RL) parameters are sensitive to the definition of REM episode. Fragmented REM episode may include intermediate S2 stage (NREM) or even short arousal (Wake) epochs; inadequate treatment will result in over-counting REM episodes.

For the purpose of this study, to evaluate a genuine REM episode (cycle) with defined onset and offset, we formulated four decision rules. These rules were formulated using our knowledge of sleep cycles and applied for both on PSG and SSA, REM estimations. Future studies are needed to evaluate the validity of these rules, especially on REM disorders, and their effect on RL and RC comparisons.

Rules:

1) REM episode starts (Onset) with three consecutive REM epochs (1.5 min);
2) REM cycle progresses if REM-fragmentation is separated by less than seven different epochs (i.e., filling the gaps).
3) The second rule applies as long as the REM episode is less than 130 epochs (65 min).
4) The distance between two REM episodes (two onsets) is higher than 100 epochs (50 min).

References

1 Biswal, S. *et al.* SLEEPNET: Automated Sleep Staging System via Deep Learning. *arXiv preprint arXiv:1707.08262* (2017).

2 Dong, H. *et al.* Mixed Neural Network Approach for Temporal Sleep Stage Classification. *IEEE Transactions on Neural Systems and Rehabilitation Engineering* (2017).

3 Supratak, A., Dong, H., Wu, C. & Guo, Y. DeepSleepNet: A model for automatic sleep stage scoring based on raw single-channel EEG. *IEEE Transactions on Neural Systems and Rehabilitation Engineering* **25**, 1998-2008 (2017).
